# Multinuclear MRI to disentangle intracellular sodium concentration and extracellular volume fraction in breast cancer

**DOI:** 10.1038/s41598-021-84616-9

**Published:** 2021-03-04

**Authors:** Carlotta Ianniello, Linda Moy, Justin Fogarty, Freya Schnabel, Sylvia Adams, Deborah Axelrod, Leon Axel, Ryan Brown, Guillaume Madelin

**Affiliations:** 1 Center for Advanced Imaging Innovation and Research (CAI2R), Department of Radiology, New York University Grossman School of Medicine, New York, NY 10016 USA; 2 Center for Biomedical Imaging, Department of Radiology, New York University Grossman School of Medicine, New York, NY 10016 USA; 3 Vilcek Institute of Graduate Biomedical Sciences, New York University Grossman School of Medicine, New York, NY 10016 USA; 4 Department of Surgery, New York University Grossman School of Medicine, New York, NY 10016 USA; 5 Department of Medicine, New York University Grossman School of Medicine, New York, NY 10016 USA; 6 Perlmutter Cancer Center, New York University Grossman School of Medicine, New York, NY 10016 USA

**Keywords:** Biomarkers, Biomedical engineering, Magnetic resonance imaging

## Abstract

The purpose of this work was to develop a novel method to disentangle the intra- and extracellular components of the total sodium concentration (TSC) in breast cancer from a combination of proton ($^{1}$H) and sodium ($^{23}\hbox {Na}$) magnetic resonance imaging (MRI) measurements. To do so, TSC is expressed as function of the intracellular sodium concentration ($\hbox {C}_{\text {IC}}$), extracellular volume fraction (ECV) and the water fraction (WF) based on a three-compartment model of the tissue. TSC is measured from $^{23}\hbox {Na}$ MRI, ECV is calculated from baseline and post-contrast $^{1}$H $\hbox {T}_{{1}}$ maps, while WF is measured with a $^{1}$H chemical shift technique. $\hbox {C}_{\text {IC}}$ is then extrapolated from the model. Proof-of-concept was demonstrated in three healthy subjects and two patients with triple negative breast cancer. In both patients, TSC was two to threefold higher in the tumor than in normal tissue. This alteration mainly resulted from increased $\hbox {C}_{\text {IC}}$ ($\sim$ 30 mM), which was $\sim$ 130% greater than in healthy conditions (10–15 mM) while the ECV was within the expected range of physiological values (0.2–0.25). Multinuclear MRI shows promise for disentangling $\hbox {C}_{\text {IC}}$ and ECV by taking advantage of complementary $^{1}$H and $^{23}\hbox {Na}$ measurements.

## Introduction

Breast cancer is the most commonly diagnosed type of cancer among women worldwide^1,2^. Breast imaging is an essential tool in breast cancer screening and diagnosis. To this day, mammography is the preferred imaging methodology used to detect breast cancer at an early stage. Other imaging techniques are often used to supplement mammography, such as ultrasound (US) and magnetic resonance imaging (MRI). Annual screening breast MRI is recommended in addition to mammography in high risk patients (i.e. patients with family history of breast cancer, BRCA gene mutation or history of chest radiation). Furthermore, breast MRI helps evaluate the extent of the disease, the response to neoadjuvant chemotherapy (NACT) and integrity of silicone implants. Dynamic contrast-enhanced (DCE) MRI is the backbone of any breast MRI protocol. DCE MRI provides the highest sensitivity for breast cancer diagnosis due to rich morphologic, tissue vascularity and pharmacokinetic information^3^. Additionally, diffusion weighted imaging (DWI) provides information on the tissue organization at the microscopic level^4^. However, all these markers are surrogates for underlying metabolic activity. As high and ultra-high field MRI have become more available, there has been a renewed interest towards X-nuclei (i.e. non hydrogen) MRI applications in breast cancer^5–8^. Unlike standard proton ($^{1}$H) MRI, X-nuclei MRI can provide functional information by probing ions involved in metabolic processes at the cellular level. This additional metabolic information could complement the morphological information offered by standard MRI.

Sodium ($^{23}\hbox {Na}$) MRI non-invasively and directly probes sodium ions which play a key role in regulating osmotic pressure and ionic homeostasis at the cellular level^9^. It has been shown that total sodium concentration (TSC) is significantly higher in malignant breast lesions compared to benign lesions and healthy fibroglandular tissue^5,8,10,11^. Further, TSC is inversely correlated to the apparent diffusion coefficient measured with diffusion MRI, whose low values are typically associated with high cellularity and malignancy^7^. While TSC can be quantified with $^{23}\hbox {Na}$ MRI, it is influenced by two main factors that confound its physiological interpretation: (1) the intracellular sodium concentration ($\hbox {C}_{\text {IC}}$), which is governed by the sodium-potassium pump, and (2) the extracellular volume (ECV) fraction, which can change with cellular swelling, death, or edema. In other words, an increase in TSC can be due to increase of the $\hbox {C}_{\text {IC}}$ or an increase of ECV with constant extracellular sodium concentration, or some combination of the two. Therefore, $\hbox {C}_{\text {IC}}$ and ECV could be more specific to cell viability, inflammation or fluid content compared to TSC alone. Unfortunately, the intrinsic low signal-to-noise ratio (SNR) associated to $^{23}\hbox {Na}$ MRI along with uncertain relaxation properties of the intra- and extra cellular sodium, make it challenging to disentangle these two contributions^12^.

Efforts have been made to isolate the intracellular sodium concentration using multiple quantum filtering (MQF) or inversion recovery (IR) $^{23}\hbox {Na}$ MRI techniques^13–15^. MQF sequences use a specific pattern of radiofrequency (RF) pulses and phase cycling to separate intra- and extracellular compartments based on differences in their biexponential $\hbox {T}_{{2}}$ relaxation properties. In biological tissues the presence and evolution of multiple quantum coherences related to restricted spin motion and tissue anisotropy can be detected with MQF sequences. The main assumption in MQF is that the isolated multiple quantum coherence signal, resulting from this filtering, originates mainly from the intracellular compartment due to its packed arrangement of proteins and molecules that restricts sodium spin motion to a higher degree compared with the extracellular environment. MQF proponents argue that by carefully selecting the amplitudes and phases of the RF pulses, it is possible to selectively isolate the triple quantum coherence component from the total sodium signal. However, the isolated signal is only a small fraction of the total signal (approximately 10%) which is already characterized by low $^{23}\hbox {Na}$ SNR^9^. Moreover, the main MQF assumption remains controversial, with many studies showing contributions of the extracellular space to the signal originating from double or triple quantum coherences^12,16–20^. Whereas MQF relies on $\hbox {T}_{{2}}$, IR techniques rely on $\hbox {T}_{{1}}$ contrast to separate the intra- and extracellular compartments: the intracellular space exhibits shorter $\hbox {T}_{{1}}$ due to its denser environment as opposed to the extracellular space and fluids which typically have longer $\hbox {T}_{{1}}$^21,22^. To separate the signals, $^{23}\hbox {Na}$ IR suppresses the fluid component, which can be subtracted from a distinct measurement of the total signal to obtain a intracellular weighted contribution.Similarly to MQF, the assumption of different $\hbox {T}_{{1}}$ between intra- and extracellular space based on the differences in the two environments is inaccurate and IR methods can only offer an “intracellular weighted” signal rather than isolating the absolute intracellular signal. Other technical drawbacks include a long, power-demanding inversion pulse that can be sensitive to $\hbox {B}_{{0}}$ and $\hbox {B}_{{1}}$ inhomogeneity^23^, along with a priori knowledge of fluid $\hbox {T}_{{1}}$. Moreover, like MQF, IR utilizes only $^{23}\hbox {Na}$ measurements, which are inherently noisy.

**Figure 1:**
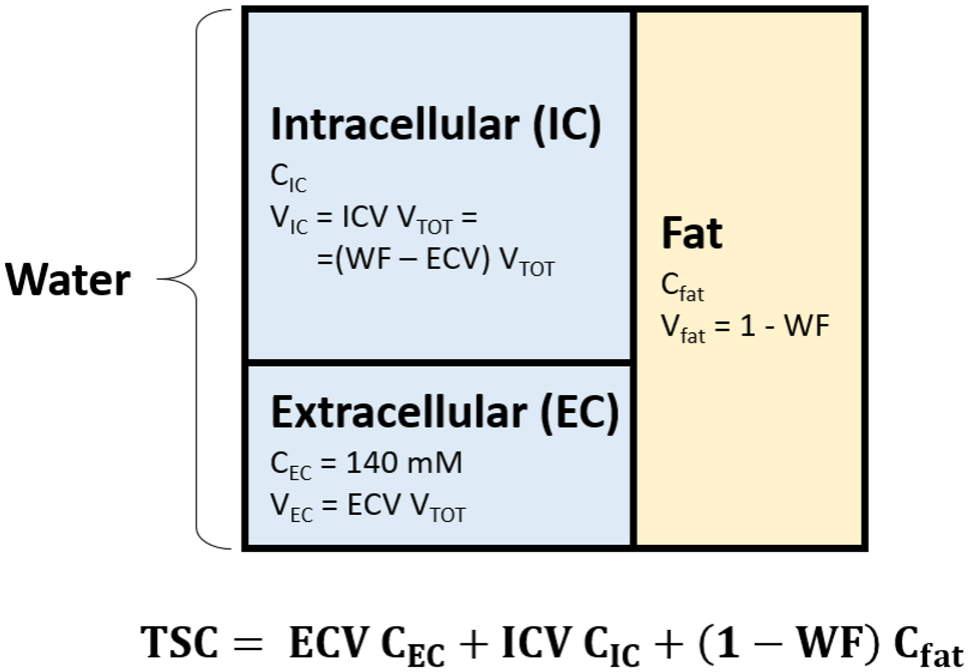
Three compartment model of an imaging voxel. Each voxel is constituted by water (blue) and fat (yellow) in different proportions. Within the water portion we identify an intracellular (IC) and an extracellular (EC) compartments. In our study we assume the extracellular sodium concentration ($\hbox {C}_{\text {EC}}$) to be fixed to 140 mM, which simplifies the calculation of intracellular sodium concentration ($\hbox {C}_{\text {IC}}$). TSC, total sodium concentration; $\hbox {C}_{\text {fat}}$, fat sodium concentration; ECV, extracellular volume fraction; ICV, intracellular volume fraction; WF, water fraction; $\hbox {V}_{\text {TOT}}$, total voxel volume.

**Figure 2:**
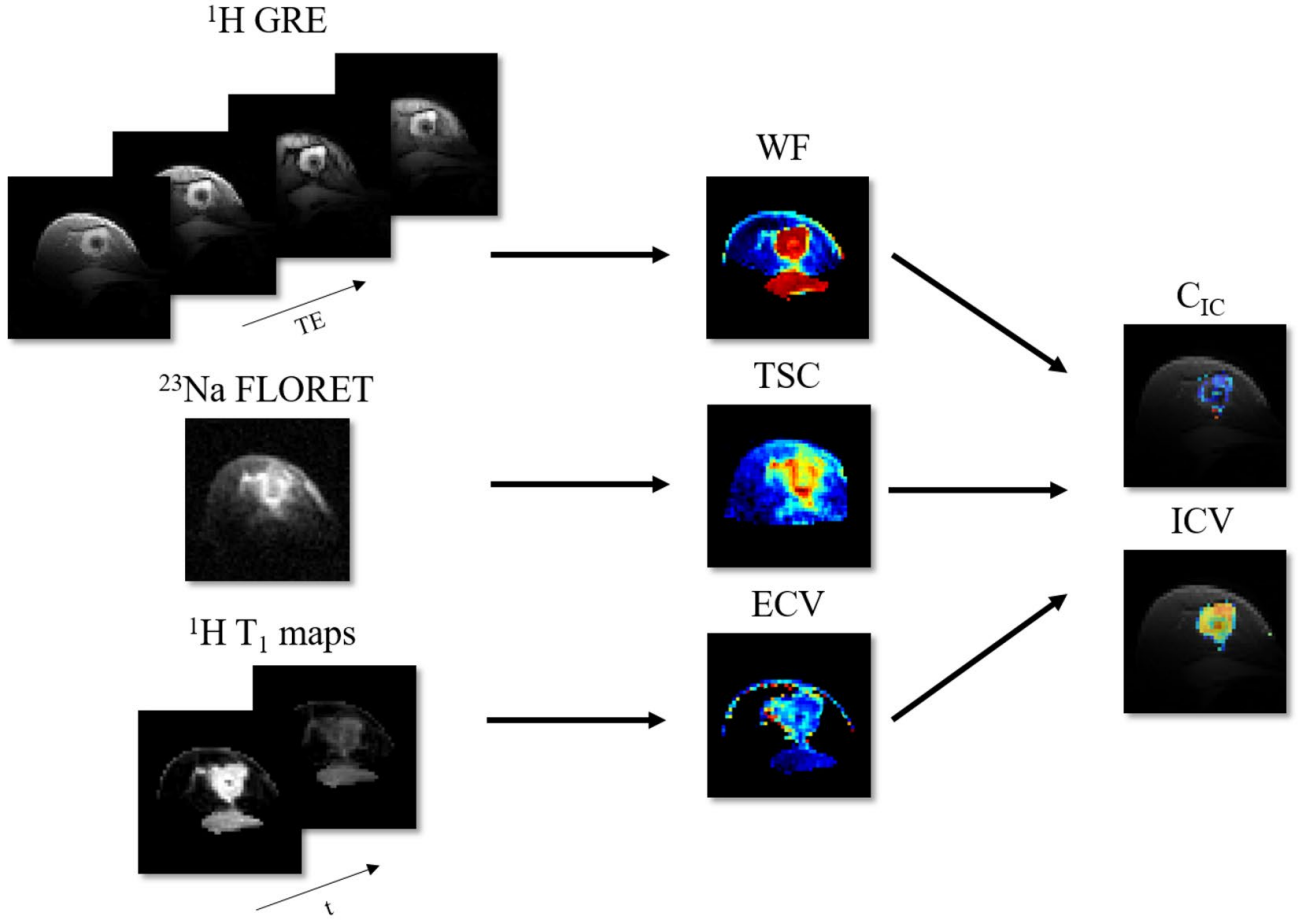
Quantification pipeline: a combination of $^{23}\hbox {Na}$ and $^{1}$H imaging modalities are employed to isolate the intracellular component of the total sodium signal. From left to right: four GRE images with incremental TE’s, a $^{23}\hbox {Na}$ FLORET image and two $^{1}$H $\hbox {T}_{{1}}$ measurements (pre and post contrast) are independently elaborated to generate, respectively, WF, TSC and ECV. Ultimately, these three maps are combined (Eq. 1) to calculate $\hbox {C}_{\text {IC}}$ and ICV.

In this study, we eliminate the need to rely on carefully calibrated RF pulses, subtle differences in relaxation times, and undiversified low-SNR $^{23}\hbox {Na}$ data. We propose a pipeline that takes advantage of a combination of $^{1}$H and $^{23}\hbox {Na}$ MRI measurements to enable $\hbox {C}_{\text {IC}}$ and ECV quantification in breast cancer. We used a three-compartment tissue model to describe the TSC in terms of components that can be measured by either $^{1}$H or $^{23}\hbox {Na}$ MRI measurements (see Fig. 1). According to the model, an imaging voxel comprises two main compartments: water and fat. The water compartment can moreover be decomposed in the intracellular and the extracellular spaces. Each compartment is characterised by its own sodium concentration and volume fraction ($\hbox {C}_{\text {IC}}$ and ICV, $\hbox {C}_{\text {EC}}$ and ECV, $\hbox {C}_{\text {fat}}$ and $\hbox {V}_{\text {fat}}$ respectively for the intracellular, extracellular and fat compartments). Summing ICV and ECV gives the water fraction (WF). While the TSC is only accessible with $^{23}\hbox {Na}$ MRI, the ECV and the WF are morphological features that can be measured via $^{1}$H MRI. By extracting ECV using contrast uptake information and WF from a chemical shift technique, we are able to determine $\hbox {C}_{\text {IC}}$. Specifically, TSC was related to $^{23}\hbox {Na}$ MRI signal intensity using a images from a multi-compartment calibration phantom with known sodium concentrations and relaxation times. ECV was measured from the ratio of the $^{1}$H $\hbox {T}_{{1}}$ change due to contrast uptake in the breast related to $^{1}$H $\hbox {T}_{{1}}$ change in a reference tissue. This method has been largely investigated in cardiac MRI to assess myocardial infarction and fibrosis^24–27^. While in cardiac applications blood is used as a reference with known volume fraction, here we chose the pectoral muscle as reference tissue due to its proximity to the breast. The WF was calculated using a four-point Dixon-based technique^28,29^. Once all other quantities in the model are measured, $\hbox {C}_{\text {IC}}$ can then be extracted from the model equation (see equation in Fig. 1). Figure 2 illustrates the pipeline in which $^{1}$H gradient echo (GRE) images, $^{1}$H $\hbox {T}_{{1}}$ maps and $^{23}\hbox {Na}$ images allows $\hbox {C}_{\text {IC}}$ quantification.

The purpose of this study was to investigate the feasibility of exploiting robust, high SNR $^{1}$H MRI to resolve the intra- and extracellular components of TSC in breast cancer from complementary $^{1}$H/$^{23}\hbox {Na}$ MRI measurements at 7 T. To demonstrate proof of concept, phantom data were collected for validation and in vivo scans were carried out on three healthy female subjects and two female patients with triple negative breast cancer (TNBC). Additional volunteers were recruited to measure TSC repeatability and $^{23}\hbox {Na}$ relaxation times.

## Results

### Phantom validation

**Figure 3:**
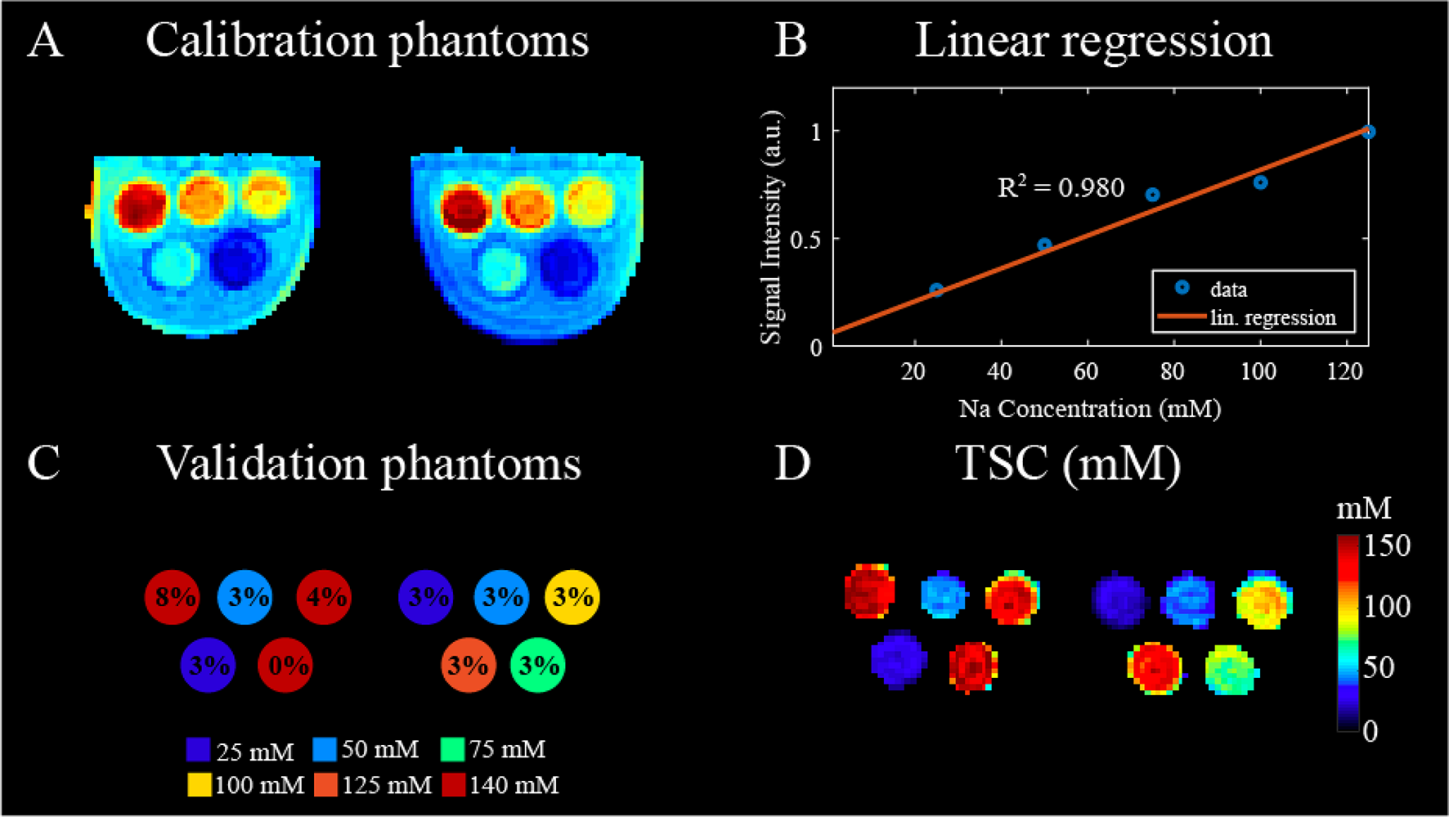
Total sodium concentration (TSC) calibration and validation in phantoms. (**A**) Calibration phantoms with ten 3% agar gels with sodium concentration ranging from 25 to 125 mM with 25 mM step. The FLORET dataset was corrected to account for $\hbox {B}_{{1}}$ profile and gels relaxation times. (**B**) Calibration curve, $\hbox {R}^2$ = 0.980. Each point in the plot corresponds to the average signal intensity of two bilateral gels with same sodium concentration. (**C**) Schematic of validation phantoms consisting of ten gels with agar concentrations between 0 and 8%, which are overlaid for each gel. The true sodium concentrations are color coded and ranged between 25 and 140 mM. (**D**) TSC map (mM) measured in validation phantoms shows good agreement with ground truth with < 7% error. Specifically, the mean TSC calculated in the 0%, 4% and 8% agar gels (true concentration: 140 mM) were, respectively, $141.9 \pm 18.4$ mM, $137.9 \pm 9.7$ mM and $150.0 \pm 12.0$ mM. In the 3% agar gels (true concentrations: 25, 50, 75, 100, 125 mM) the mean TSC were: $26.5 \pm 2.9$ mM, $47.3 \pm 3.8$ mM, $78.3 \pm 6.3$ mM, 95.5 ± 7.6 mM and $125.4 \pm 10.0$ mM. The relaxation times of the 0%, 3%, 4% and 8% agar gels are reported in Supplementary Table S1.

TSC calibration and quantification in phantoms are illustrated in Fig. 3. A good linear fit ($\hbox {R}^{2}$ = 0.980) was found between signal intensities and sodium concentrations in ten 3% agar gels with known $^{23}\hbox {Na}$ concentrations and relaxation properties that constituted the calibration phantoms (Fig. 3A,B). The TSC map calculated in the validation phantoms, consisting of ten agar gels with agar concentrations ranging from 0 to 8% and sodium concentrations ranging from 25 to 140 mM, showed good agreement with the true sodium concentration of the gels (Fig. 3C,D). The absolute error between the measured and true concentrations in all the gels was less than 7%.

### In vivo results

**Figure 4:**
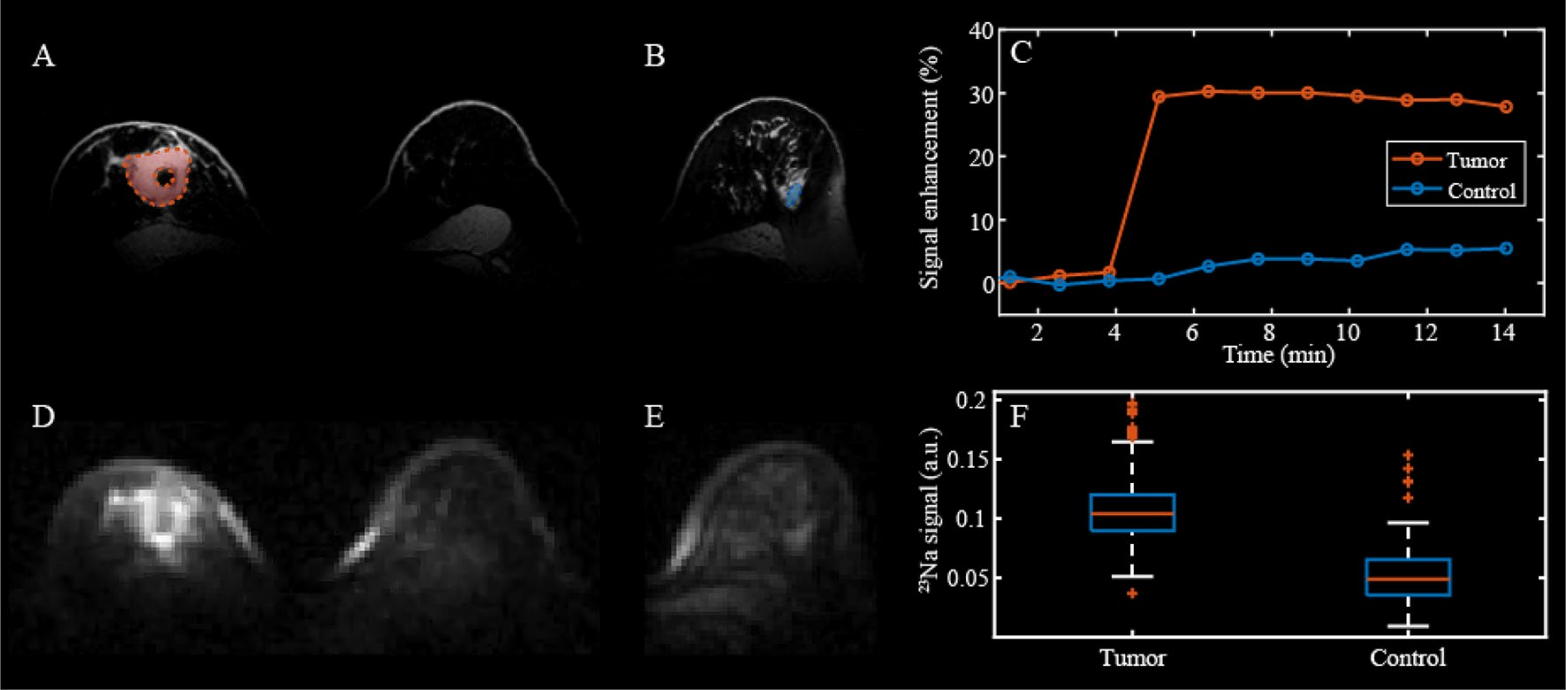
MRI images in 51-year-old patient with triple negative breast cancer. (**A**) Proton $\hbox {T}_{{1}}$w fat suppressed (FS) anatomical image. The area in red is the ROI containing the lesion. (**B**) Different slice of the same volume showing the control ROI (blue region) in the contralateral breast. (**C**) Dynamic $^{1}$H signal enhancement shows high uptake of contrast agent (CA) in a 645-voxel-ROI that includes the tumor (red ROI in panel A) compared to a 100-voxel control ROI in contralateral healthy fibroglandular tissue (blue region in panel **B**) (**D**) Sodium image, corresponding to the $^{1}$H $\hbox {T}_{{1}}$w in panel A, acquired with a FLORET sequence showing hyperintensity in the tumor. (**E**) Sodium image in which healthy contralateral tissue is visible, co-registered with the $^{1}$H $\hbox {T}_{{1}}$w image in (**B**). (**F**) Boxplot showing the distribution of $^{23}\hbox {Na}$ signal within the tumor and in the contralateral healthy fibroglandular tissue (control). Sodium signal intensity in the lesion is 2-fold higher than in the control.

Images from a postmenopausal TNBC patient are shown in Fig. 4. The high resolution $^{1}$H $\hbox {T}_{{1}}$w image (Fig. 4A) reveals a $\sim$ 3 cm lesion in the right breast which is also visible in the corresponding $^{23}\hbox {Na}$ image (Fig. 4D). The hypointense circle within the lesion is attributable to the biopsy marker. The coil provided good coverage of the breast and pectoral muscle, which was crucial for the ECV measurement. The red area in Fig. 4A is a 645-voxel-ROI containing the lesion. Figure 4B shows the control ROI drawn in blue in the contralateral breast (Fig. 4E shows the correspondent slice in the $^{23}\hbox {Na}$ volume). Due to the high fat content in the contralateral breast, only 24 adjacent voxels were selected in the control ROI.

As expected, the dynamic signal enhancement (Fig. 4C) shows higher contrast agent uptake in the lesion compared to healthy control tissue. Similarly, the sodium signal is 2-fold higher in the lesion compared to healthy fibroglandular tissue (Fig. 4F).

**Figure 5:**
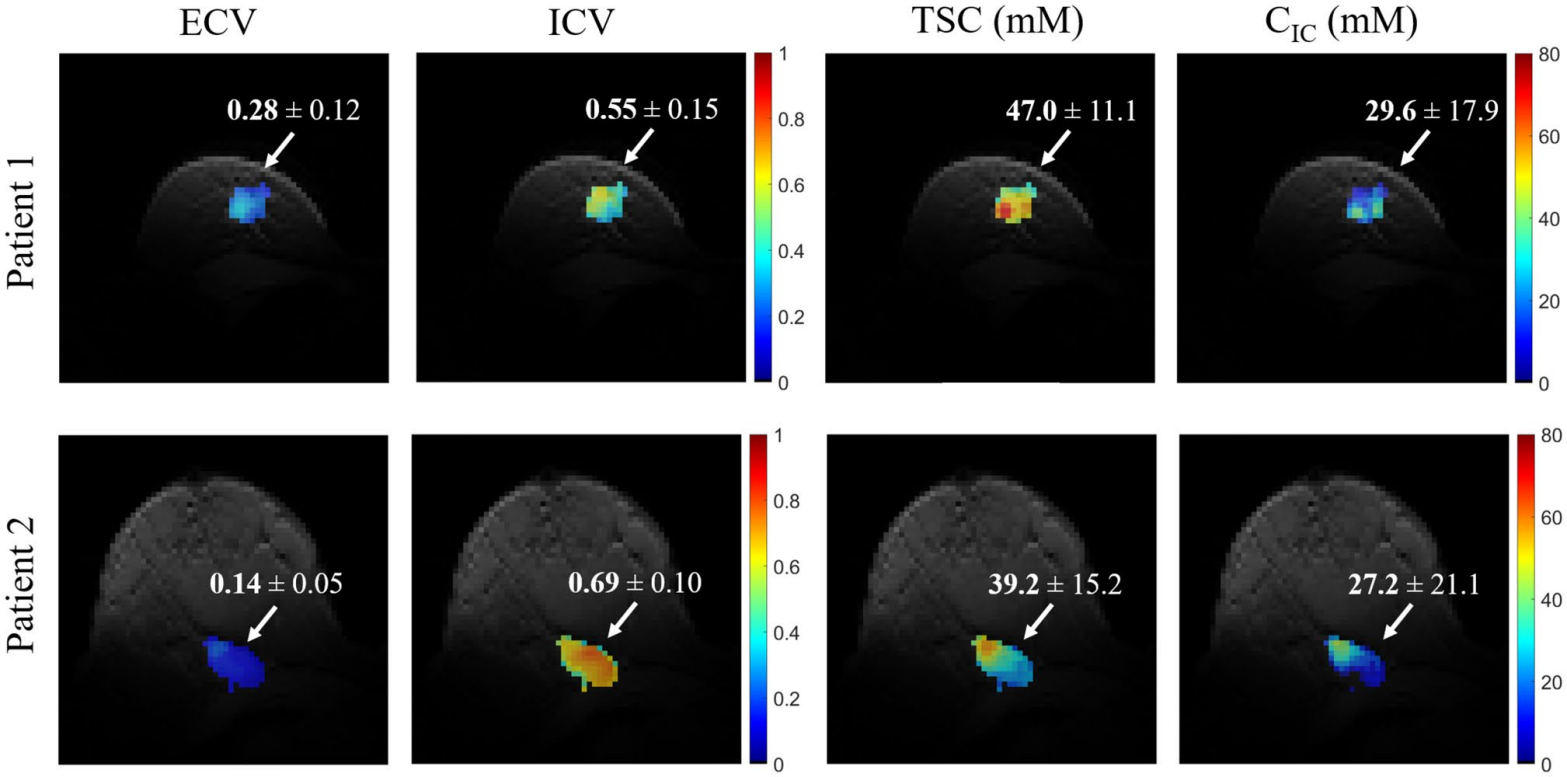
Quantitative maps in a post-menopausal 51-years-old patient with triple negative breast cancer (Patient 1) and in a post-menopausal 58-years-old patient with triple negative breast cancer (Patient 2). In patient 1: average ECV and ICV in the lesion were respectively $0.28 \pm 0.12$ and $0.55 \pm 0.15.$ TSC in the lesion was 47.0 ± 11.1 mM, which was approximately double the TSC measured in contralateral healthy glandular tissue ($23.0 \pm 7.6$ mM, not visible in the figure) in accordance with previous findings. $\hbox {C}_{\text {IC}}$ in the lesion was $29.6 \pm 17.9$ mM. In patient 2: average ECV and ICV in the lesion were respectively $0.14 \pm 0.05$ and 0.69 ± 0.10. TSC in the lesion was $39.2 \pm 15.2$ mM, which was three times higher than in contralateral healthy tissue ($13.5 \pm 7.5$ mM). $\hbox {C}_{\text {IC}}$ in the lesion was $27.2 \pm 21.1$ mM. ECV, extracellular volume fraction; ISC, intracellular volume fraction; TSC, total sodium concentration; $\hbox {C}_{\text {IC}}$, intracellular sodium concentration.

Quantification maps in two TNBC patient are reported in Fig. 5. In both patients an increase in TSC was observed in the tumor compared to contralateral healthy glandular tissue, which was in accordance with previous findings^5,7,8^ (Patient 1: tumor: 47.0 ± 11.1 mM, healthy fibroglandular tissue: $22.9 \pm 7.6$ mM; Patient 2: tumor: $39.2 \pm 15.2$ mM, healthy fibroglandular tissue: $13.5 \pm 7.5$ mM). Specifically, in one patient (top row in Fig. 5) the TSC, $\hbox {C}_{\text {IC}}$, ECV and ICV in a 645 adjacent voxels 3D ROI containing the lesion were respectively 47.0 ± 11.1 mM, $29.6 \pm 17.9$ mM, $0.28 \pm 0.12$, $0.55 \pm 0.15.$ In a second patient (bottom row in Fig. 5) the TSC, $\hbox {C}_{\text {IC}}$, ECV and ICV in a 575 adjacent voxels 3D ROI containing the lesion were respectively 39.2 ± 15.2 mM, $27.2 \pm 21.1$ mM, $0.14 \pm 0.05,$ $0.69 \pm 0.10.$

**Figure 6:**
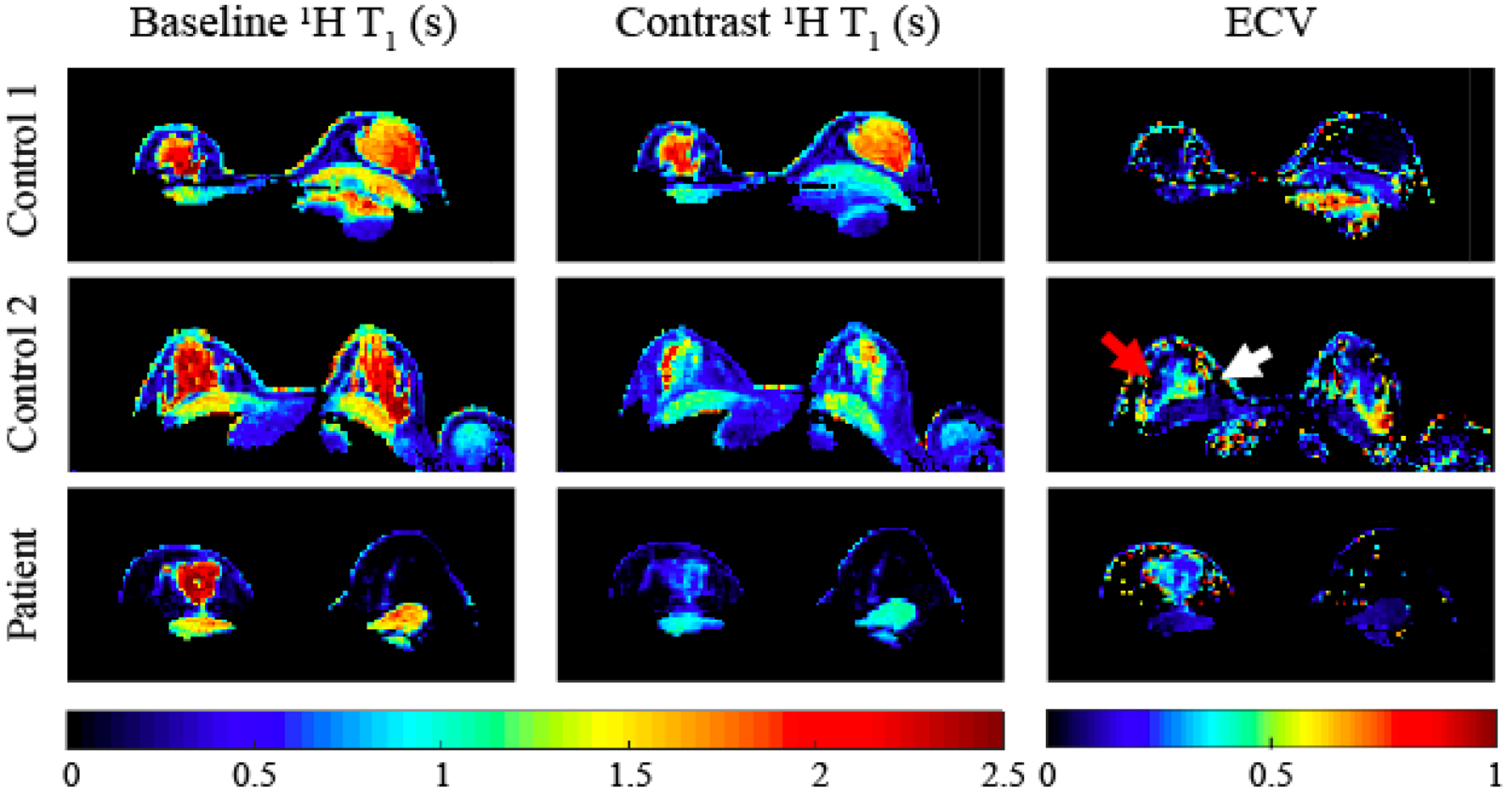
Baseline $\hbox {T}_{{1}}$, post contrast $\hbox {T}_{{1}}$ and ECV in two healthy controls and a patient. In control 1 (top row) poor CA uptake with only 6% $\hbox {T}_{{1}}$ decrease and consequently extremely low average ECV (0.06 ± 0.10) were observed. In control 2 (center row) the average $\hbox {T}_{{1}}$ decrease in the fibrograndular tissue was 42% but with a non-uniform pattern, resulting in regions with higher ECV of 0.29 ± 0.24 (white arrows) where the uptake was greater, and regions with lower ECV of $0.03 \pm 0.03$ in regions with lower uptake. The $\hbox {T}_{{1}}$ decrease in the lesion of a 51-years-old triple negative breast cancer patient (bottom row) was 67% which resulted into mean ECV in the lesion of $0.28 \pm 0.12.$

We observed a range of ECV values in our cohort. Baseline and post contrast $\hbox {T}_{{1}}$ in a 34-years-old healthy subject (Fig. 6, top row) show poor contrast agent uptake with only 6% $\hbox {T}_{{1}}$ decrease and consequently low average ECV ($0.06 \pm 0.10$). In a 27-years-old healthy subject the ECV varied from $0.29 \pm 0.24$ in high uptake regions to 0.03 ± 0.03 in low uptake regions (Fig. 6, center row). In comparison, the $\hbox {T}_{{1}}$ decrease in the lesion of the TNBC patient was 67%, which resulted in ECV = $0.28 \pm 0.12$ (Fig. 6, bottom row), while the contralateral healthy tissue had 5% $\hbox {T}_{{1}}$ reduction and ECV = 0.02 ± 0.02.

The TSC in healthy fibroglandular tissue measured in the cohort of ten healthy subjects was $33.6 \pm 5.2,$ which was in agreement with previous findings^5–8^.

### Error propagation

Physiological variations of $\hbox {C}_{\text {EC}}$ and ECV in the pectoral muscle can affect ECV and $\hbox {C}_{\text {IC}}$ quantification in the lesion as reported in Table 1. In summary, ± 7% $\hbox {C}_{\text {EC}}$ uncertainty results in ± 14% $\hbox {C}_{\text {IC}}$ variation, while ± 17% $\hbox {ECV}_{\text {ref}}$ translates to ± 17% ECV and ± 26% $\hbox {C}_{\text {IC}}$ variations. Table 2 shows how inaccurate $^{23}\hbox {Na}$ relaxation times affect TSC and $\hbox {C}_{\text {IC}}$ quantification. Variations of ±  50% of $\hbox {T}_{{1}}$, $\hbox {T}_{2,s}$ and $\hbox {T}_{2,l}$ result in variation of TSC of, respectively, $\pm 14$%, ±  15% and ±  0.03% and in variation of $\hbox {C}_{\text {IC}}$ of, respectively, ±  39%, ±  33% and ±  1%. Errors in the relative percentages of the fast and slow components of the transverse relaxation (here assumed to be 60:40) can lead to quantification errors. Specifically, ± 30% error in the fast component fraction leads to ± 8.5% error in TSC and ± 24.3% error in $\hbox {C}_{\text {IC}}$. Moreover, including or omitting the fat compartment in the model in Fig. 1 also affects the quantification. For the TNBC patient in Fig. 4, $\hbox {C}_{\text {IC}}$ in the lesion ROI was 29.6 ± 7.7 mM when the fat compartment was included in the voxel model and 32.8 ± 19.7 mM when it was excluded.

## Discussion

Here we present a new method that utilizes $^{1}$H MRI to disentangle the intra- and extracellular contributions of the TSC. Unlike MQF and IR, this method does not rely on differences in relaxation times between the intra- and extracellular spaces. Relaxation times of these two compartments have been measured in animal models using chemical shift reagents which offset the resonance frequency of sodium in the extracellular space, allowing to isolate the sodium signal from intracellular space^30^. However, these reagents are toxic and therefore not suitable for human studies. Moreover the differences in relaxation between the two compartments are very subtle, which makes signal separation challenging^12^. Furthermore, other techniques aim at isolating the intracellular sodium signal using only $^{23}\hbox {Na}$ MRI^31^, which can be challenging due to its low intrinsic SNR.

We take advantage of high SNR $^{1}$H MRI measurements and use them to complement $^{23}\hbox {Na}$ MRI. Specifically, we defined a three compartment model, described by Fig. 1, where TSC is a function of WF and ECV, which can be measured via $^{1}$H MRI. WF is calculated from $^{1}$H GRE acquisitions using the IDEAL method, which is highly precise and robust against $\hbox {B}_{{0}}$ and $\hbox {B}_{{1}}$ inhomogeneities^24^. ECV is calculated from baseline and post-contrast $^{1}$H $\hbox {T}_{{1}}$ maps measured with MP2RAGE acquisitions which are independent of $\hbox {B}_{1}^{-}$, $\hbox {T}^{*}_{2}$, proton density and, with the proper selection of flip angles, largely independent from $\hbox {B}_{1}^{+}$^32^.

In two TNBC patients, we found elevated TSC in the lesion compared to normal tissue, in agreement with previous findings^5,7,8,10^. Moreover, in both patients we found that $\hbox {C}_{\text {IC}}$ was $\sim$130% greater than what is reported in literature for healthy tissue (10–15 mM) while the ECV was within the physiological values (approximately 0.2)^9,33,34^. These findings suggest that the increase in TSC mainly originated from increase in the intracellular sodium, which could be due to sodium-potassium pump dysfunction^9,35^.

Although this feasibility study was limited to three healthy subjects and two TNBC patients, contrast agent uptake varied widely with breast density and morphology in healthy tissue. As expected, increased permeability of blood vessels and cellular metabolic activity resulted in a rapid and large uptake in cancerous tissue. While healthy tissues are expected to show slower and less pronounced uptake, we observed almost zero uptake in subsections of healthy tissues in measurements performed 10 minutes after injection. The lack of uptake results in artificially low ECV, which makes cross-sectional study difficult. Moreover, the breast perfusion, and consequently the measured ECV, can vary during the menstrual cycle in pre-menopausal women due to estrogen fluctuation^36–38^. Increase of contrast enhancement is observed during weeks 1 and 4, while week 2 is typically preferred to perform the MRI examinations. Unfortunately, the menstrual status of the three healthy subjects in this study was not available. Nonetheless, this technique could be suitable for longitudinal cancer studies, where contrast agent uptake is anticipated in the tissue of interest within a reasonable injection-to-measurement time period. One such application is assessing tumor response to therapy. While current gold standard MRI methods for assessing tumor response to therapy are based on monitoring changes in the tumor structure (i.e. tumor size) which can take weeks to manifest, $^{23}\hbox {Na}$ MRI registers changes in cellular metabolism, which are a precursor to structural change^35^. This can be beneficial for monitoring cancer response to treatment at an early stage, potentially allowing tailored therapy planning. Moreover, differentiating between intra- and extracellular contributions may infer new physiological information related to ion homeostasis. Further, it is reasonable to assume that model parameters such as pectoral muscle ECV and $\hbox {C}_{\text {EC}}$, whose error propagation were explored, will remain stable over time and therefore will not confound relative tumor ECV and $\hbox {C}_{\text {IC}}$.

The proposed method, although free from assumptions regarding differences between the intra- and extracellular $^{23}\hbox {Na}$ relaxation times, still relies on a reasonable estimate of $^{23}\hbox {Na}$ $\hbox {T}_{{1}}$ and $\hbox {T}_{{2}}$ in the lesion to correct for relaxation effects in TSC quantification. Despite being straightforward, measuring global $^{23}\hbox {Na}$ relaxation times is very time consuming (on the order of 60 minutes for each relaxation time, depending on the desired imaging parameters). Since subject specific measurements are infeasible, we applied the relaxation correction factor $\lambda$ that was calculated from $\hbox {T}_{{1}}$ and $\hbox {T}_{{2}}$ measurements in 3 subjects (Supplementary figures S5, S6, S7, S8 and S9).

However, an alteration in relaxation properties in the tumor due to changes in its molecular environment will affect TSC and, consequently, $\hbox {C}_{\text {IC}}$ quantification. The use of an ultra-short TE sequence such as FLORET mitigates this effect, especially in the eventuality of increased $\hbox {T}_{{2}}$ in the lesion. Reversely, a decrease of $\hbox {T}_{{2}}$ in the lesion could lead to underestimation of TSC with our method. In this study similar $\hbox {T}_{{1}}$ and $\hbox {T}_{{2}}$ were observed in one healthy volunteer and two patients with fibroadenoma. Nonetheless, relaxation times of a larger patient cohort need to be measured in order to better assess potential variability across subjects and tissue types. The development of fast $^{23}\hbox {Na}$ $\hbox {T}_{{1}}$ and $\hbox {T}_{{2}}$ quantification methods would allow to correct for patient-specific $^{23}\hbox {Na}$ relaxation times, making TSC and $\hbox {C}_{\text {IC}}$ quantification more accurate. Unfortunately, due to sodium’s low SNR, this area of research is still largely unexplored. Another drawback of this technique is the injection of contrast agent. While gadolinium-based contrast agents are used routinely in the clinic, recent work has shown that deposition in bone and brain tissue that may accumulate with multiple administrations^39^. In the context of the technique presented here, it remains to be determined whether the prospect of revealing metabolic response to treatment outweighs the burden of multiple contrast agent injections.

In conclusion, we presented the proof of concept of a new method to disentangle the intra- and extracellular components of TSC in breast cancer. This method is based on direct $^{23}\hbox {Na}$ MRI measurement of TSC complemented with $^{1}$H MRI measurements of water fraction and $\hbox {T}_{{1}}$ relaxation before and after contrast enhancement. These robust, high SNR $^{1}$H scans show promise for replacing delicate filtering methods that rely on low SNR $^{23}\hbox {Na}$ MRI data and prior knowledge of $^{23}\hbox {Na}$ relaxation time in cellular compartments. The proposed multi-nuclear MRI method could inform on early tumor response to therapy and potentially provide clues on underlying metabolic activity in different tumor phenotypes.

**Table 1:** Error propagation due to uncertainty on $\hbox {C}_{\text {EC}}$ and ECV of muscle. On the left side the mean $\hbox {C}_{\text {IC}}$ in an ROI containing the lesion (Patient 1 in Fig. 5) is reported for different values of $\hbox {C}_{\text {EC}}$. On the right side mean ECV and $\hbox {C}_{\text {IC}}$ in the lesion are reported for different values of the muscle ECV. In the last row the mean uncertainty, defined as mean percentage variation relative to the central values, is reported. The values of $\hbox {C}_{\text {EC}}$ and muscle ECV we used in our method and the corresponding mean $\hbox {C}_{\text {IC}}$ and ECV in the lesion are highlighted in bold. Notations are: $\hbox {C}_{\text {EC}}$ extracellular sodium concentration, $\hbox {C}_{\text {IC}}$ intracellular sodium concentration, ECV extracellular volume fraction.

	Input	Output	Input	Output	
	${{C}}_{{EC}}$ (mM)	Lesion ${C}_{{{IC}}}$ (mM)	Muscle ECV	Lesion ECV	Lesion ${C}_{{{IC}}}$ (mM)
	130	30.6	0.10	0.25	33.5
	135	28.8	0.11	0.28	30.5
	**140**	**26**.**8**	**0**.**12**	**0**.**30**	**26**.**8**
	145	24.8	0.13	0.33	23.0
	150	22.9	0.14	0.35	19.3
Mean uncertainty	± 7%	± 14%	± 17%	± 17%	± 26%

**Table 2:** Error propagation from uncertainty of $^{23}\hbox {Na}$ relaxation times. In the left section the mean TSC and $\hbox {C}_{\text {IC}}$ in an ROI containing the lesion (Patient 1 in Fig. 5) is reported for different values of $^{23}\hbox {Na}$ $\hbox {T}_{{1}}$. In the central section mean TSC and $\hbox {C}_{\text {IC}}$ in the lesion are reported for different values of $\hbox {T}_{2,s}$. in the rightmost section mean $\hbox {C}_{\text {IC}}$ and TSC of the tumor are reported for a range of $\hbox {T}_{2,l}$. In the last row the mean uncertainty, defined as mean percentage variation relative to the central values, is reported. The relaxation times that we set in our method and the corresponding mean quantification results in the lesion are highlighted in bold. Due to the scarcity of literature references for ^23^Na relaxation times in breast cancer, an abundant range of relaxation properties (± 50%) was simulated. Notations are: $\hbox {C}_{\text {IC}}$ intracellular sodium concentration, TSC total sodium concentration.

	Input	Output		Input	Output		Input	Output	
	${T}_{{1}}$ (ms)	Lesion TSC (mM)	Lesion ${C}_{{{IC}}}$ (mM)	${T}_{{{2,s}}}$ (ms)	Lesion TSC (mM)	Lesion ${C}_{{{IC}}}$ (mM)	${{T}}_{{{2,l}}}$ (ms)	Lesion TSC (mM)	Lesion ${C}_{{{IC}}}$ (mM)
	16	41.2	18.5	0.25	57.2	43.8	8	47.3	30.2
	24	43.6	22.3	0.38	50.3	33.5	12	47.1	29.8
	**32**	**47**.**0**	**29**.**6**	**0**.**5**	**47**.**0**	**29**.**6**	**16**	**47**.**0**	**29**.**6**
	40	50.9	34.6	0.63	44.9	24.4	20	47.0	29.6
	48	55.1	41.7	1	41.9	22.2	24	47.0	29.6
Mean uncertainty	± 50%	± 14%	± 39%	± 50%	± 15%	± 33%	± 50%	± 0.03%	± 1%

## Methods

### Three-compartment model

A simplified three-compartment model of breast tissue, as shown in Fig. 1, was used to disentangle the intracellular and extracellular components of the TSC. In our model, a voxel is composed of two main compartments: water (blue) and fat (yellow). Within the water compartment, which includes the breast fibroglandular tissue as well as tumor tissue, we defined an intracellular and an extracellular space. According to this model, TSC can be broken into three contributions:

1 $$\begin{aligned} TSC = ECV \cdot C_{EC} + ICV \cdot C_{IC} + (1 - WF) \cdot C_{fat} \end{aligned}$$

where $\hbox {C}_{\text {IC}}$, $\hbox {C}_{\text {EC}}$, and $\hbox {C}_{\text {fat}}$ are respectively the intracellular, extracellular and the fat sodium concentrations, WF is the water fraction, ECV is the extracellular volume fraction and ICV = WF – ECV is the intracellular volume fraction. Although fat has low sodium content (of the order of 10 mM)^40^, it is an important component in the model because fat-fibroglandular interfaces, which are abundant in the breast, can cause an overestimation of $\hbox {C}_{\text {IC}}$ in some voxels due to partial volume effect.

### Total sodium concentration

TSC was directly measured from $^{23}\hbox {Na}$ images using a phantom calibration method similar to that described by other authors^7,41–43^. The relationship between signal intensity and sodium concentration was determined using two identical calibration phantoms that were placed into each side of a bilateral breast coil. Each phantom contained five 3% agar gels with known sodium concentrations ranging from 25 to 125 mM (with increase step of 25 mM) immersed in a water and salt bath (50 mM) (Fig. 3A). The relaxation effects, which for simplicity are assumed to be purely quadrupolar, were accounted for by applying to the signal intensities the correction factor $\lambda = \frac{1 - e^{-{TR/T_{1}} }}{1-cos(FA) e^{-{TR/T_{1}}}} \cdot (0.6 e^{-{TE/T_{2,s}}} + 0.4 e^{-{TE/T_{2,l}}})$^12^, where FA, TE and TR are the flip angle, echo time and repetition time of imaging sequence. $\hbox {T}_{{1}}$ is the gel longitudinal relaxation time that was measured separately by fitting a monoexponential function to signals acquired with multiple TRs (Supplementary Figure S1), while $\hbox {T}_{2,s}$ and $\hbox {T}_{2,l}$ are respectively the short and long transverse relaxations times that were measured by fitting a biexponential function to signals acquired with multiple TEs (Supplementary Fig. S2). In the Supplementary Information more details on the gel $^{23}\hbox {Na}$ relaxation time measurements and fitting results and errors are provided. Secondly, we corrected for $\hbox {B}_{{1}}$ inhomogeneity by normalizing the gel phantom images by a smoothed image acquired in a large uniform phantom that filled the coil^44^. This approximation relies on the assumption that at low frequency (78.6 MHz at 7 T), the sodium $\hbox {B}_{{1}}$ field is minimally perturbed by the presence of a dielectric load^45^. Partial volume effects characteristic of low resolution $^{23}\hbox {Na}$ MRI were mitigated by deconvolving the images with the point spread function of the sequence using the built-in deconvblind function in MATLAB^®^ R2018b (MathWorks, Natick, MA). The point spread function was calculated by applying the reconstruction algorithm to a uniform k-space weighted by the bioexponential $\hbox {T}_{{2}}$ decay. Further details on PVC assessment and the effect of $\hbox {T}_{{2}}$ relaxation on the PSF can be found in the Supplementary Information (Supplementary Figures S3 and S4).

To evaluate the phantom calibration method, we performed measurements in a validation phantom with multiple compartments containing known $^{23}\hbox {Na}$ concentrations. The compartments consisted of three 140 mM concentration gels with agar concentration of 0%, 4% and 8% along with seven out of the ten 3% agar gels used for calibration rearranged in different locations. Different agar concentrations were used to generate gels with different relaxation times, which were measured following the same procedure described above. While in the calibration phantom the sodium gels were immersed in a liquid bath, the validation gels were directly inserted into the coil without any liquid bath. This allowed us to assess the TSC quantification accuracy in a different loading condition.

To determine TSC in vivo, we applied the same correction steps as above, except the $^{23}\hbox {Na}$ relaxation times were set to those of fibroglandular tissue which were measured in one healthy subject and two fibroadenoma patients (Supplementary Figures S5, S6, S7, S8 and S9) ($\hbox {T}_{2,s}$= 0.5 ms, $\hbox {T}_{2,l}$= 15 ms, $\hbox {T}_{{1}}$ = 37 ms), and were in accordance with the literature^46^. Details on the $^{23}\hbox {Na}$ relaxation time measurement and fitting results and errors are provided in the Supplementary Information.

Repeatability of the TSC measurement was tested on three healthy female subjects and the results are shown in the Supplementary Information (Supplementary Figure S10). Briefly, variation of TSC measured within the same session and in separate session was less then 1.17 standard deviations.

### Water fraction

Water fraction was calculated by processing a set of $^{1}$H 3D-VIBE datasets with four TEs using a hierarchical IDEAL method^28,47^ available in the ISMRM water-fat separation toolbox^29^. IDEAL is a Dixon-based water/fat separation method which consists of acquiring three or more images, each one with a different relative phase between the water and fat signals. This offers greater robustness to $\hbox {B}_{{0}}$ and $\hbox {B}_{{1}}$ inhomogeneities compared to the classic 2-points Dixon method^48^. By explicitly measuring water fraction, we easily identified voxels comprised predominantly by fat tissue. This allowed us to define the sodium concentration in fat ($\hbox {C}_{\text {fat}}$) according to the average TSC value in voxels in which the WF was below 10%.

### Extracellular volume fraction

Calculation of the ECV from contrast-enhanced and native $^{1}$H $\hbox {T}_{{1}}$ measurements has been largely investigated in cardiac MRI to quantitatively characterize myocardial infarction and fibrosis^24–27^. We applied the same principle to breast tissue, in which contrast agent (CA) causes $\hbox {T}_{{1}}$ reduction from baseline in proportion to the contrast agent concentration according to $1/\Delta T_{1} = \Delta R_{1} = r_{1} \cdot [CA]$, where $R_1$ is relaxation rate and $r_1$ the contrast agent relaxivity. Notably the contrast agent uptake provides insight on the underlying cellular makeup because it circulates only in the extracellular space. We can directly retrieve the pixel-wise ECV by normalizing the relaxation rate change between pre and post contrast agent injection in the breast by that in a reference tissue^49^:

2 $$\begin{aligned} ECV = ECV_{ref} \frac{R_{1,post} - R_{1,pre}}{R_{1,post,ref} - R_{1,pre,ref}} \end{aligned}$$

whereas cardiac applications utilize blood as the reference tissue, we used the pectoral muscle ($\hbox {ECV}_{\text {ref}}$ = 0.12) because it is well characterized in the literature^50–53^ and its proximity to the breast allowed it to be imaged in a local coil. However, due to its location, the pectoral muscle area is affected by reduced $\hbox {B}_{{1}}$ which could potentially affect the $\hbox {T}_{{1}}$ measurement. Phantom experiments were carried out to assess this effect (Supplementary Figure S11).

### Intracellular sodium concentration

Once WF, TSC and ECV are calculated, $\hbox {C}_{\text {IC}}$ can be simply derived from Eq. (1) under the assumption that $\hbox {C}_{\text {EC}}$ is fixed in our model and equal to 140 mM^9,54^. Figure 2 illustrates the pipeline used to calculate $\hbox {C}_{\text {IC}}$ (and ICV) from the combination of $^{1}$H WF, $^{23}\hbox {Na}$ images and baseline and post-contrast $^{1}$H $\hbox {T}_{{1}}$.

### Imaging protocol

All MRI measurements were performed on a Siemens 7 T MAGNETOM scanner (Siemens Healthineers, Erlangen, Germany) using an in-house built transmit/receive dual-tuned bilateral breast coil^55^ with two proton and eight sodium receive channels. The coil was constituted by two shielded unilateral units, one for each breast, each one comprising one $^{1}$H transmit/receive volume coil, one $^{23}\hbox {Na}$ transmit volume coil and a four-channel $^{23}\hbox {Na}$ receive array, all arranged in a nested fashion. The New York University Office of Science and Research Institutional Review Board approved this Health Insurance Portability and Accountability Act study and all participants were scanned after providing informed consent. All experiments were performed in accordance with the relevant institutional and national guidelines. We scanned 3 healthy female subjects (mean age: $32 \pm 4$ years, age range: 27–35 years) and two breast cancer patients (age 51 and 58 years old, both postmenopausal) diagnosed with >3cm triple negative breast cancer (TNBC). Ten additional healthy subjects (mean age: $30 \pm 7$ years, age range: 22–44 years) were scanned without contrast injection to only measure TSC. Of these ten, three subjects were enrolled in a repeatability study (see Supplementary Information). One further healthy subject (29 years old) and two patients with fibroadenoma (28 and 29 years old) were recruited to measure $^{23}\hbox {Na}$ relaxation times (see Supplementary Figures S5–S9).

After running a localizer, $\hbox {B}_{{0}}$ shim adjustments and calibrating the excitation reference voltages, a $^{1}$H $\hbox {T}_{{1}}$-weighted fat suppressed (FS) 3D-VIBE acquisition was run for anatomical reference^56^. Following, four 3D-VIBE datasets with different TE’s were acquired to calculate the water fraction using the IDEAL method. Sodium images were then acquired using a fermat looped orthogonally encoded trajectories (FLORET) sequence^57^. Sodium FLORET images were acquired prior to Gd injection to ensure that the sodium signal was not affected by the contrast agent. Proton $\hbox {T}_{{1}}$ maps were acquired using a Magnetization Prepared 2 Rapid Acquisition Gradient Echoes (MP2RAGE) sequence^32^ at baseline and 10 minutes after contrast agent injection (0.1ml/kg of Gadobutrol, Gadavist, Bayer HealthCare, Whippany, NJ). During this time DCE $^{1}$H $\hbox {T}_{{1}}$w FS 3D-VIBE images with 76.5 s temporal resolution were acquired in order to record signal change due to contrast agent perfusion. Four baseline time points were acquired prior contrast agent injection. The total exam duration was approximately 47 minutes. The acquisition parameters were set to the following values:

- **High resolution FS 3D-VIBE for anatomical reference**: TR = 7.0 ms, TE = 2.14 ms, FA = 4$^{\circ }$, voxel size = 0.9 $\times$ 0.9 $\hbox {mm}^{2}$, slice thickness = 1.0 mM, acquisition time = 3:11.
- **3D-VIBE for water fraction calculation**: TR = 7.0 ms, TE = 2.04/2.24/2.44/2.64 ms, FA = 6$^{\circ }$, voxel size = 1.4 $\times$ 1.4 $\hbox {mm}^{2}$, slice thickness = 4.0 mm, total acquisition time = 4:16.
- **MP2RAGE for** $^{\mathbf{1 }}\mathbf{H }$ $\mathbf{T} _\mathbf{1 }$ **map and ECV calculation**: TR = 4000.0 ms, TE = 1.27 ms, TI - 0.7/2.5 ms, FA = 4/5$^{\circ }$, voxel size = 1.9 $\times$ 1.9 $\hbox {mm}^{2}$, slice thickness = 1.1 mm, acquisition time = 5:29.
- **DCE GRE for contrast uptake assessment**: TR = 7.0 ms, TE = 2.14 ms, FA = 4$^{\circ }$, voxel size = 1.4 $\times$ 1.4 $\hbox {mm}^{2}$, slice thickness = 1.5 mm, number of measurements = 12, total acquisition time = 15:18.
- **FLORET for** $^\mathbf{23 }\mathbf{Na }$ **imaging and TSC quantification**: TR = 60.0 ms, TE = 0.2 ms, FA = 80$^{\circ }$, number of hubs/angle = 3/45$^{\circ }$, number of interleaves (per hub) = 400, number of averages = 8, voxel size = 2.8 $\times$ 2.8 $\times$ 2.8 $\hbox {mm}^{3}$, acquisition time = 9:36.

For displaying purposes, fibroglandular tissue and the tumors were segmented out by selecting voxels in the breast with WF>50%.

### Error propagation

As mentioned in the previous sections, in our model we fixed $\hbox {C}_{\text {EC}}$ to 140 mM and $\hbox {ECV}_{\text {ref}}$ to 0.12. Variability of these quantities can lead to deviations in other parameters. To assess error propagation from $\hbox {C}_{\text {EC}}$ variations, the quantification was performed for a range of $\hbox {C}_{\text {EC}}$ (= 130–150 mM) while fixing $\hbox {ECV}_{\text {ref}} = 0.12$. Similarly, to assess error propagation from $\hbox {ECV}_{\text {ref}}$ variations, the quantification was performed for $\hbox {ECV}_{\text {ref}}$ = 0.10–0.14 with fixed $\hbox {C}_{\text {EC}}$ = 140 mM. Such ranges were chosen based on literature findings^58–63^. The mean uncertainties on the final concentrations were calculated as $\pm \frac{1}{2} \cdot \frac{\Delta C}{C_0}$, where $\Delta C$ is the width of output concentration interval and $C_{0}$ is the center of the interval. Similarly, error propagation from misestimations of $^{23}\hbox {Na}$ global relaxation times was explored by varying one among $\hbox {T}_{{1}}$  $\hbox {T}_{2,s}$ and $\hbox {T}_{2,l}$ while fixing the other two. Due to the scarcity of literature references on $^{23}\hbox {Na}$ relaxation times in breast cancer, an abundant range of relaxation properties (± 50%) was simulated. For sake of simplicity in this study we assumed pure quadrupolar $^{23}\hbox {Na}$ relaxation with relative percentage of the fast and slow components of transverse relaxation of 60:40. Errors related to this assumption were investigated by considering different ratios. In order to assess the impact of the fat compartment on $\hbox {C}_{\text {IC}}$  we compared results with and without the compartment.

## Supplementary information

Supplementary Information.
