# Supplementary material for: Multinuclear MRI to disentangle intracellular sodium concentration and extracellular volume fraction in breast cancer
Source: Sci Rep. 2021 Mar 4;11:5156. doi: 10.1038/s41598-021-84616-9 (PMC7933187; doi:10.1038/s41598-021-84616-9)
Supplement: Supplementary file 1 — Supplementary Information. [file 41598_2021_84616_MOESM1_ESM.pdf]

# Multinuclear MRI to disentangle intracellular sodium concentration and extracellular volume fraction in breast cancer

**Carlotta Ianniello , Linda Moy , Justin Fogarty , Freya Schnabel , Sylvia Adams , Deborah Axelrod , Leon Axel , Ryan Brown , and Guillaume Madelin***

*Guillaume.Madelin@nyulangone.org

## Supplementary Information

### Phantoms $^{23}$Na relaxation times

The $^{23}$Na longitudinal relaxation time $T_1$ of the agar gels used for the calibration and validation phantoms were measured using the saturation recovery method by fitting the monoexponential function $S_0 \cdot (\sin(FA)(1-e^{-TR/T_1}))/(1-\cos(FA)e^{-TR/T_1})$ to the signals acquired with a FLORET sequence with multiple repetition times. In the equation, $S_0$ is a scaling factor proportional to the spin density, FA is the flip angle, TR is the repetition time and $T_1$is the longitudinal relaxation time. At each TR, the signal in each phantom was averaged in a 3D ROI of approximately 500 voxels in order to improve the fitting. The sequence parameters used to measure $T_1$ in the 3% agar gels in the calibration and validation phantom were: TE = 0.2 ms, TR = 25, 30, 40, 50, 75, 100, 125, 150, 200, 300, 500 ms, FA = 90°, rectangular pulse duration = 1 ms, number of hubs/angle = 3/45°, number of interleaves per hub = 175, number of averages = 3, voxel size = 3.8 × 3.8 × 3.8 mm$^3$. The sequence parameters used to measure $T_1$ of the 0, 4, and 8% gels in the validation phantom were: TE = 0.2 ms, TR = 14, 20, 25, 30, 35, 40, 45, 50, 60, 80, 100, 125, 150, 200, 300, 500 ms, FA = 90°, rectangular pulse duration = 0.8 ms, number of hubs/angle = 3/45°, number of interleaves per hub = 450, number of averages = 1, voxel size = 3.8 × 3.8 × 3.8 mm$^3$. Fitting curves for the four gels are shown in Supplementary Figure S1. For all fitting curves $R^2 > 0.99$ and RMSE $< 0.0042$.

The $^{23}$Na transverse relaxation times $T_{2,l}$ and $T_{2,s}$ of the agar gels were measured by fitting the monoexponential function (for the liquid phantoms) $S_0 \cdot e^{-TE/T_2}$ or the biexponential function (for the gel phantoms) $S_0 \cdot (Ae^{-TE/T_{2,s}} + (1-A)e^{-TE/T_{2,l}})$ to the signals acquired with FLORET sequence with multiple echo times. In the equations $S_0$ is a scaling factor proportional to the spin density, TE is the echo time, $T_2$ is the transverse relaxation time in liquid, $T_{2,l}$ and $T_{2,s}$ are the long and short components of the transverse relaxation time in gels and A is the weighting factor of the short decay component. As for the $T_1$ measurement, the signal in each phantom was averaged in 3D ROIs of approximately 500 voxels. The sequence parameters used to measure $T_{2,l}$ and $T_{2,s}$ in the 3% agar gels used in the calibration and validation phantom were: TR = 250 ms, TE = 0.2, 0.4, 0.6, 0.8, 1, 1.5, 2, 4, 8, 15, 30, 50, 100 ms, FA = 90°, rectangular pulse duration = 1 ms, number of hubs/angle = 3/45°, number of interleaves per hub = 175, number of averages = 3, voxel size = 3.8 × 3.8 × 3.8 mm$^3$. The sequence parameters used to measure $T_{2,l}$ and $T_{2,s}$ of the 0, 4, and 8% gels used in the validation phantom were: TR = 250ms, TE = 0.2, 0.4, 0.6, 0.8, 1, 1.3, 1.6, 2, 2.5, 3, 4, 5, 10, 15, 20, 30, 40, 50, 114 ms, FA = 90°, rectangular pulse duration = 0.8 ms, number of hubs/angle = 3/45°, number of interleaves per hub = 200, number of averages = 1, voxel size = 3.8 × 3.8 × 3.8 mm$^3$. Fitting curves are shown in Supplementary Figure S2. For all fitting curves $R^2 > 0.99$ and RMSE $< 0.0061$. The relaxation times of all phantom are reported in the table below (Supplementary Table S1):

| Gel | $T_1$ (ms) | $T_{2,l}$ (ms) | $T_{2,s}$ (ms) |
|---|---|---|---|
| 0% Agar | 54.9 | 48.1 | 48.1 |
| 3% Agar | 36.9 | 22.6 | 4.8 |
| 4% Agar | 36.4 | 25.4 | 5.3 |
| 8% Agar | 29.5 | 21.9 | 4.0 |

**Supplementary Table S1.** $^{23}$Na relaxation times of 0, 3, 4 and 8% agar gels used in TSC phantom.

### Partial volume correction assessment

Partial volume correction was performed by deconvolving the $^{23}$Na image by the point spread function (PSF) of the FLORET sequence. To assess the effect of partial volume correction on image resolution, a phantom was built by placing an insert

constituted by 5 rows of glass rods with diameters ranging from 1 to 5 mm and spacing equal to their diameters in a dome shaped container filled with 52 mM saline solution. The resolution phantom was placed in one side of the breast coil, while the other side was loaded with a second dome container filled only with a 52 mM saline solution, without the glass rods insert. FLORET images of the phantoms were acquired with the following sequence parameters: TR = 60.0 ms, TE = 0.2 ms, FA = 80°, number of hubs/angle = 3/45°, number of interleaves (per hub) = 400, number of averages = 8, voxel size = 2.8 × 2.8 × 2.8 $mm^3$, acquisition time = 9:36 (Supplementary Figure S3). Total sodium concentration (TSC) maps were calculated with and without deconvolution. 1D profiles of TSC across the glass rods show that PVC improved TSC quantification near the rods-bath interfaces. The effect of $T_2$ decay on the PSF was evaluated by comparing the PSF calculated with and without the bioexponential T2 decay factor $0.6 \cdot e^{-t/T_{2,s}} + 0.4 \cdot e^{-t/T_{2,l}}$ where $T_{2,l}$ = 15 ms and $T_{2,s}$ = 0.5 ms which are representative of transverse relaxation times in healthy fibroglandular tissue. Supplementary Figure S4 shows that $T_2$ decay correction had negligible impact on the PSF.

### In vivo $^{23}$Na relaxation times

Sodium relaxation times were measured in one healthy volunteer and two patients with fibroadenoma.

The $T_1$ map was obtained by voxelwise fitting a monoexponential function to the signal acquired with a FLORET sequence with multiple TRs (saturation recovery method, similarly to the phantom $T_1$ measurement). While the healthy subject was scanned in two separate sessions, one to measure $T_1$ and one to measure $T_2$ in the two patients both measurement were performed within the same session. Therefore, in order to keep the total scan time within about one hour, less time points were acquired. For the healthy subject the sequence parameters were: TR = 20, 24, 35, 45, 60, 75, 100, 120, 150, 300 ms, TE = 0.1 ms, FA = 70°, rectangular pulse duration = 1 ms, number of hubs/angle = 3/45°, number of interleaves (per hub) = 250, number of averages = 4, voxel size = 3.8 × 3.8 × 3.8 $mm^3$. For the patients the sequence parameters were the same except for the reduced number of TR = 24, 35, 60, 100, 300 ms. Supplementary Figure S5 shows the $T_1$ map in the fibroglandular tissue of the healthy subject superimposed over a coregistered $^1$H image, the distribution of $T_1$ in the fibroglandular tissue and the fitting curve in a 118-voxel-ROI. The mean $T_1$ in the fibroglandular tissue was 37.1 ± 10.6 ms. Similarly, Supplementary Figure S6 shows the $T_1$ in the fibroglandular tissue of one fibroadenoma patient superimposed over a coregistered $^1$H image. The red arrow corresponds to the position of the biopsy clip which was located within the lesion. The figure also shows the $T_1$ distribution in the fibroglandular tissue and the fitting curve in a 50-voxel-ROI. The mean $T_1$ across the whole fibroglandular tissue was 32.7 ± 7.2 ms. Overall, no difference in $T_1$ relaxation was observed between the lesion and healthy contralateral tissue: in the lesion $T_1$ = 34.7 ± 3.4 ms, in healthy contralateral tissue $T_1$ = 34.6 ± 4.0 ms.

$T_{2,l}$ and $T_{2,s}$ maps were obtained by voxelwise fitting a biexponential function to signals acquired with a FLORET sequence with multiple TEs (as in the phantom measurement). Similarly to the $T_1$ measurement, fewer data points were acquired in the two patients due to the total scan time limitation. For the healthy subject the sequence parameters were: TR = 120 ms, TE = 0.1, 0.2, 0.4, 0.6, 0.8, 1, 1.5, 2, 4, 8, 16, 32, 48 ms, FA = 90°, rectangular pulse duration = 1 ms, number of hubs/angle = 3/45°, number of interleaves (per hub) = 175, number of averages = 4, voxel size = 3.8 × 3.8 × 3.8 $mm^3$. For the patients all sequence parameters were kept the same except for the reduced number of TE = 0.1, 0.2, 0.4, 0.6, 0.8, 1.5, 8, 48 ms. Supplementary Figure S7 shows $T_{2,l}$ and $T_{2,s}$ maps in the healthy subject superimposed over a coregistered $^1$H image and their distribution in the fibroglandular tissue, along with the biexponential fitting curve in a 102-voxel hand-drawn ROI in the fibroglandular tissue. $T_{2,l}$ and $T_{2,s}$ in the fibroglandular tissue were, respectively, 15.6 ± 3.4 ms and 0.54 ± 0.21 ms with 59% contribution of the short component. Supplementary Figure S8 shows the $T_{2,l}$ and $T_{2,s}$ maps, distribution and fitting curve in the fibroglandular tissue of one fibroadenoma patient. The red arrow indicates the location of the lesion. The mean $T_{2,l}$ and $T_{2,s}$ in the whole fibroglandular tissue were, respectively, 14.8 ± 5.1 ms and 0.45 ± 0.16 ms. In the lesion $T_{2,l}$ = 15.9 ± 5.7 ms and $T_{2,s}$ = 0.48 ± 0.13 ms, in healthy contralateral tissue $T_{2,l}$ = 18.2 ± 2.1 ms and $T_{2,s}$ = 0.42 ± 0.11 ms.

Supplementary Figure S9 shows the distributions of $^{23}$Na relaxation properties in the fibroglandular tissue of all three subjects (one healthy, two with fibroadenoma). On the y-axis the normalized number of occurrences is represented. For all relaxation times the three distribution are in good agreement, showing little change in $T_1$, $T_{2,l}$ and $T_{2,s}$. While a substantial change of relaxation times in the lesion would likely result in two separate peaks in the distributions, in all distributions only one single peak is observed.

### TSC repeatability

Repeatability of the TSC measurement was investigated in a cohort of three healthy female subjects (mean age = 26 ± 4 years, range: 22-29 years old). For each subject three TSC measurements in two separate sessions were performed. Measurements 1 and 2 were acquired within the same session using the same shim settings and patient position. Following a few minutes break, during which the subjects were taken out of the scanner, measurement 3 was performed after repositioning the subjects and readjusting the shim. For each measurement four 3D-VIBE datasets with different TEs were acquired in order to calculate the water fraction using the IDEAL method which was used to segment the fibroglandular tissue (TR = 7.0 ms, TE = 2.04/2.24/2.44/2.64 ms, FA = 6°, voxel size = 1.4 × 1.4 $mm^2$, slice thickness = 4.0 mm, total acquisition time = 4:16). For the

TSC measurements the sequence parameters were the same used in the final protocol: TR = 60.0 ms, TE = 0.2 ms, FA = 80°, number of hubs/angle = 3/45°, number of interleaves (per hub) = 400, number of averages = 8, voxel size = 2.8 × 2.8 × 2.8 $mm^3$, acquisition time = 9:36. The repeated TSC measurements were analyzed using the Bland-Altman method by plotting the difference between each pair of measurements (measurements 1-2, 2-3 and 1-3) versus the mean of each pair of measurements (Supplementary Figure S10). The difference between TSC acquired within the same session (measurement 1-2) was less than 1.17 standard deviations. The difference between TSC acquired in separate sessions (measurement 1-3 and measurement 2-3) was less than 1.13 standard deviations. In the Bland-Altman plot in Supplementary Figure S10 each pair of measurements is identified by a different color (blue: measurement 1-2, red: measurement 1-3, green: measurement 2-3), the circles refer to measurements within the same session (intra-assay) while the stars refer to measurements performed in separate sessions (inter-assay).

### $^1$H $T_1$ measurement assessment

The proposed method necessitates measuring $^1$H $T_1$ in the pectoral muscle, which lays outside of the breast coil cavities. Therefore, it was important to assess the effect of reduced $B_1^+$ on the MP2RAGE $T_1$ measurement. A two-compartment phantom (water-based compartment in the inside and oil bath in the outside) was placed in the breast coil so that roughly half of the phantom was laying outside of the coil cavities. Co-registered flip angle (FA) and $^1$H $T_1$ maps were acquired using respectively:

- $^1$H $T_1$ map: MP2RAGE, TR = 5000.0 ms, TE = 1.18 ms, TI - 0.7/2.5 ms, FA = 4/5°, voxel size = 2.6 × 2.6 $mm^2$, slice thickness = 1.9 mm, acquisition time = 4:51.
- FA map: TR = 17430.0 ms, TE = 1.94 ms, FA = 5°, voxel size = 2.8 × 2.8 $mm^2$, slice thickness = 4 mm, acquisition time = 00:44.

Supplementary Figure S11 shows that while the FA profile decreases by 33% over a 57 mm range in the phantom moving towards the outside of the cavity, $T_1$ is uniform within 10% of its maximum in the same range. In our experience, the pectoral muscle lays between 0 and 40 mm outside of the coil. In this range, $T_1$ is within 10% of its maximum between 0 and 32 mm outside of the coil and drops by 25% between 32 and 40 mm.

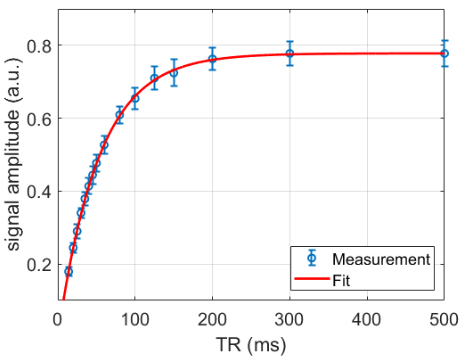


**(a)** $T_1$ fitting - 0% agar gel.
$T_{1,0\%}$ = 54.9 ms, $R^2$ = 0.9997, RMSE = 0.0038

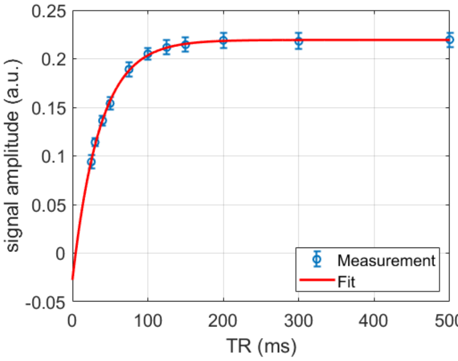


**(b)** $T_1$ fitting - 3% agar gel.
$T_{1,3\%}$ = 36.9 ms, $R^2$ = 0.9988, RMSE = 0.0019

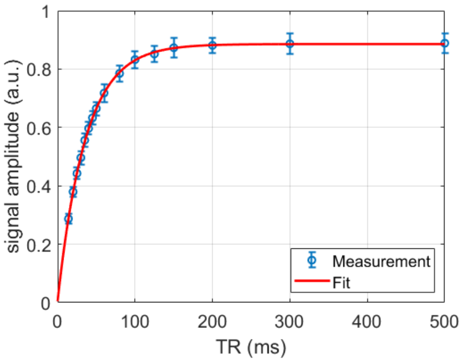


**(c)** $T_1$ fitting - 4% agar gel.
$T_{1,4\%}$ = 36.4 ms, $R^2$ = 0.9998, RMSE = 0.0032

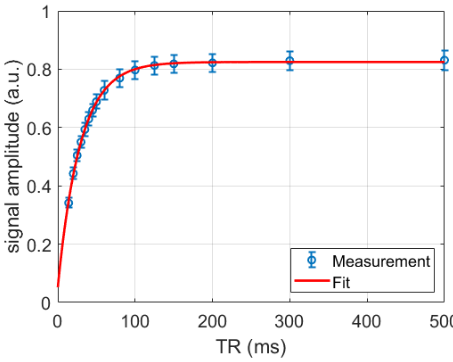


**(d)** $T_1$ fitting - 8% agar gel.
$T_{1,8\%}$ = 29.5 ms, $R^2$ = 0.9994, RMSE = 0.0042

**Supplementary Figure S1.** Phantoms $T_1$ fitting curves. $^{23}$Na $T_1$ relaxation times of the calibration and validation phantoms were measured by fitting a monoexponential function to the signals acquired with FLORET sequence with multiple TRs ranging from 14 ms to 500 ms. At each time point, the signal was averaged in a 3D ROI of approximately 500 voxels in order to increase SNR and consequently improve the fitting.

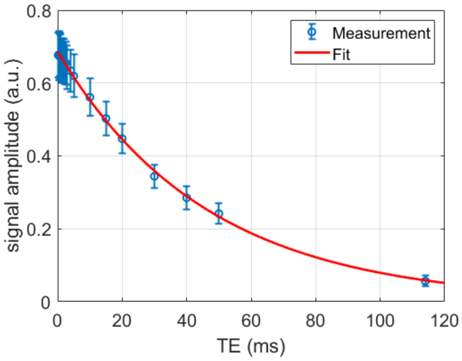


**(a)** $T_2$ fitting - 0% agar gel.
$T_{2,0\%}$ = 48.1 ms,
$R^2$ = 0.9990, RMSE = 0.0061

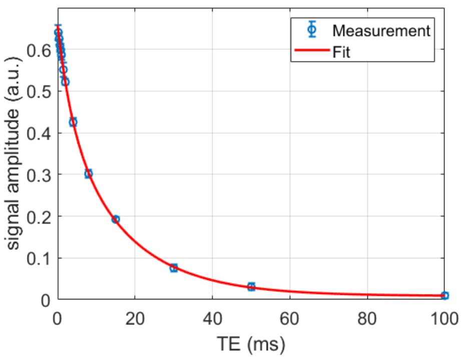


**(b)** $T_2$ fitting - 3% agar gel.
$T_{2l,3\%}$ = 22.6 ms, $T_{2s,3\%}$ = 4.8 ms,
$R^2$ = 0.9999, RMSE = 0.0026

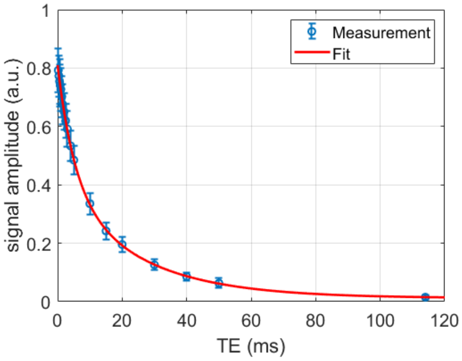


**(c)** $T_2$ fitting - 4% agar gel.
$T_{2l,4\%}$ = 25.4 ms, $T_{2s,4\%}$ = 5.3 ms,
$R^2$ = 0.9999, RMSE = 0.0026

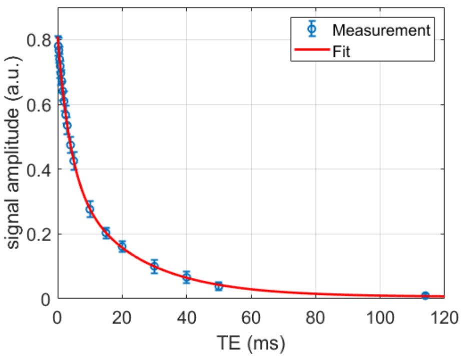


**(d)** $T_2$ fitting - 8% agar gel.
$T_{2l,4\%}$ = 21.9 ms, $T_{2s,4\%}$ = 4.0 ms,
$R^2$ = 0.9999, RMSE = 0.0027

**Supplementary Figure S2.** Phantoms $T_2$ fitting curves. $^{23}$Na $T_{2,l}$ and $T_{2,s}$ relaxation times of the calibration and validation phantoms were measured by fitting a monoexponential (for the 0% agar phantom) or biexponential (for the 3, 4 and 8% agar phantoms) function to the signals acquired with FLORET sequence with multiple TEs ranging from 0.2 ms to 114 ms. As for $T_1$ measurements, at each time point the signal was averaged in a 3D ROI of approximately 500 voxels.

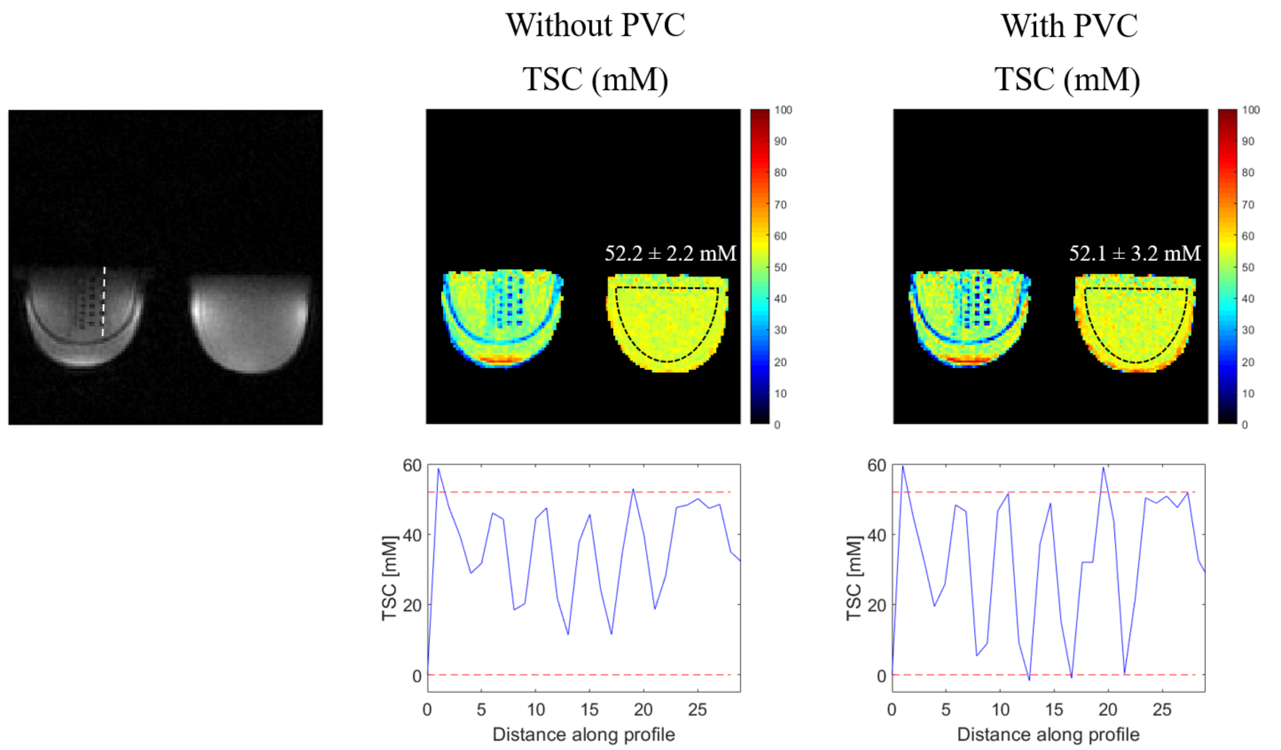


**Supplementary Figure S3.** Partial volume correction assessment in a resolution phantom. FLORET $^{23}$Na image (top left) shows the resolution phantoms design: both left and right dome-shaped phantoms were filled with 52 mM phosphate buffered saline solution, the right phantom also included an insert constituted by 5 rows of glass rods with diameters ranging between 1 and 5 mm and spacing equal to their diameters. TSC maps were calculated without (top center) and with (top right) PVC. TSC profiles across the 5-mm glass rods (dashed line in the FLORET image) are plotted for the non PVC (bottom center) and PVC (bottom right) case. The TSC profiles show a better quantification result when PVC is applied where the TSC is closer to 0 in correspondence with the glass rods and to 52 mM in correspondence to the saline solution.

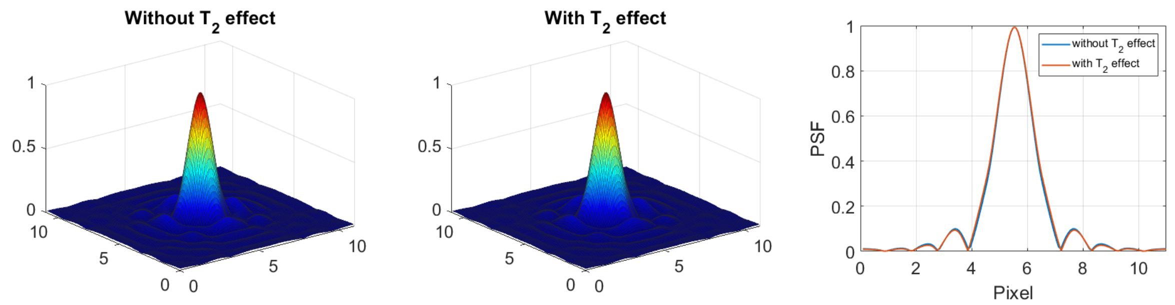


**Supplementary Figure S4.** Point spread function calculated without (left) and with (center) the contribution of $T_2$ relaxation decay (with $T_{2,l}$ = 15 ms and $T_{2,s}$ = 0.5 ms). 1D profile across the center of the PSF (right) shows great similarity between the two PSFs. In both cases the full width at half maximum (FWHM) was approximately 1.5 voxels.

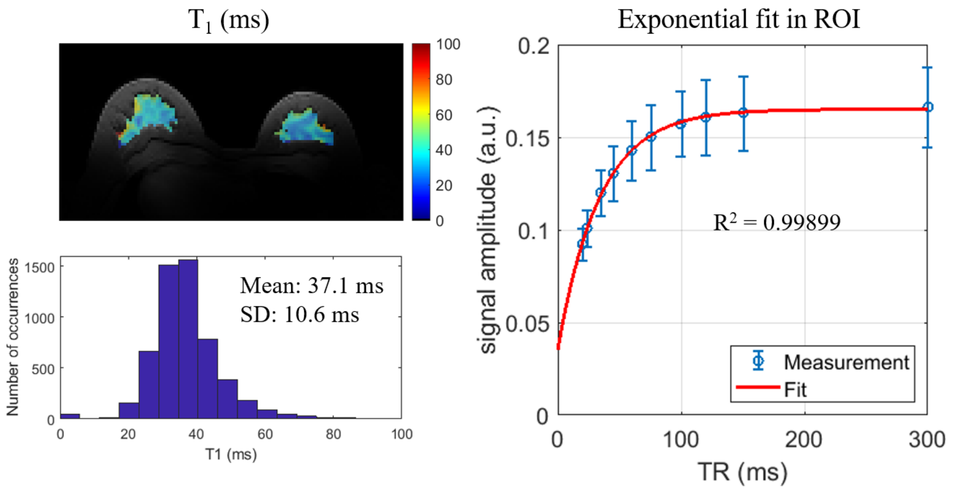


**Supplementary Figure S5.** In vivo $^{23}$Na $T_1$ map superimposed over $^{1}$H T1w images and corresponding distribution within the fibroglandular tissue in a 29-year-old healthy subject. Bioexponential fitting curve in a 118-voxel hand-drawn ROI in the fibroglandular tissue shows good fit to the data with $R^2 > 0.99$. In the fibroglandular tissue $T_1 = 37.1 \pm 10.6$ ms, which was in accordance with literature findings.

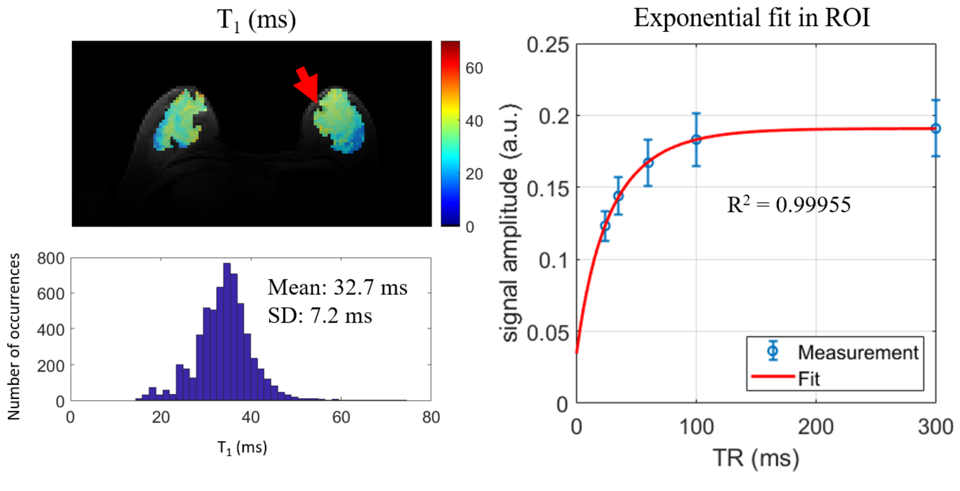


**Supplementary Figure S6.** In vivo $^{23}$Na $T_1$ map superimposed over $^{1}$H T1w images and corresponding distribution within the fibroglandular tissue in a 28-year-old patient with a fibroadenoma. Bioexponential fitting curve in a 50-voxel hand-drawn ROI in the fibroglandular tissue shows good fit to the data with $R^2 > 0.99$. In the fibroglandular tissue $T_1 = 32.7 \pm 7.2$ ms. The lesion (indicated by red arrow in the $T_1$ map) didn't show different $T_1$ properties compared to the rest of the healthy fibroglandular tissue: in an ROI containing the lesion $T_1 = 34.7 \pm 3.4$ ms, while in healthy contralateral tissue $T_1 = 34.6 \pm 4.0$ ms.

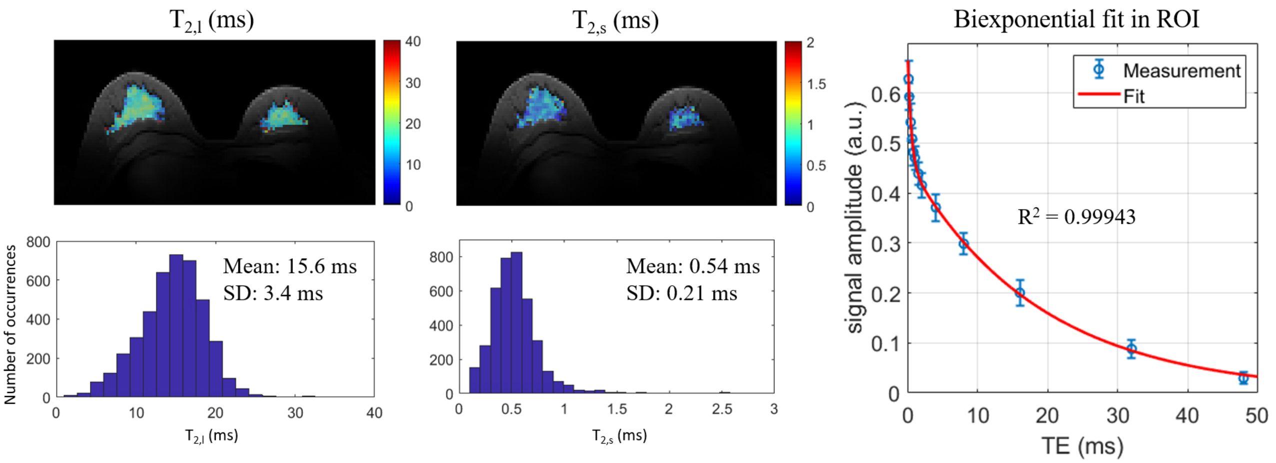


**Supplementary Figure S7.** In vivo [23]Na $T_{2,l}$ and $T_{2,s}$ maps superimposed over [1]H T1w images and corresponding distribution within the fibroglandular tissue in a 29-year-old healthy subject. Bioexponential fitting curve in a 102-voxel hand-drawn ROI in the fibroglandular tissue shows good fit to the data with $R^2 > 0.99$. The measured transverse relaxation times were $T_{2,l}$ = 15.6±3.4 ms and $T_{2,s}$ = 0.54 ± 0.21 ms, with 59% contribution of the short component, which was in accordance with literature findings.

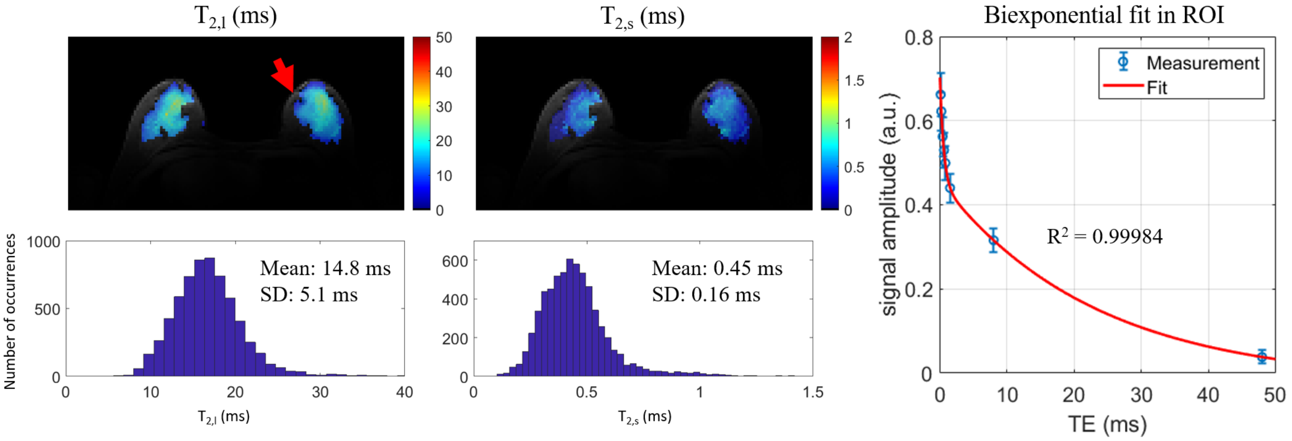


**Supplementary Figure S8.** In vivo [23]Na $T_{2,l}$ and $T_{2,s}$ maps superimposed over [1]H T1w images and corresponding distribution within the fibroglandular tissue in a 28-year-old patient with fibroadenoma. Bioexponential fitting curve in a 50-voxel hand-drawn ROI in the fibroglandular tissue shows good fit to the data with $R^2 > 0.99$. The measured transverse relaxation times were $T_{2,l}$ = 14.8±5.1 ms and $T_{2,s}$ = 0.45 ± 0.16 ms. In the lesion (indicated by red arrow in the $T_{2,l}$ map) $T_{2,l}$ = 15.9 ± 5.7 ms and $T_{2,s}$ = 0.48 ± 0.13 ms, in healthy contralateral tissue $T_{2,l}$ = 18.2 ± 2.1 ms and $T_{2,s}$ = 0.42 ± 0.11 ms.

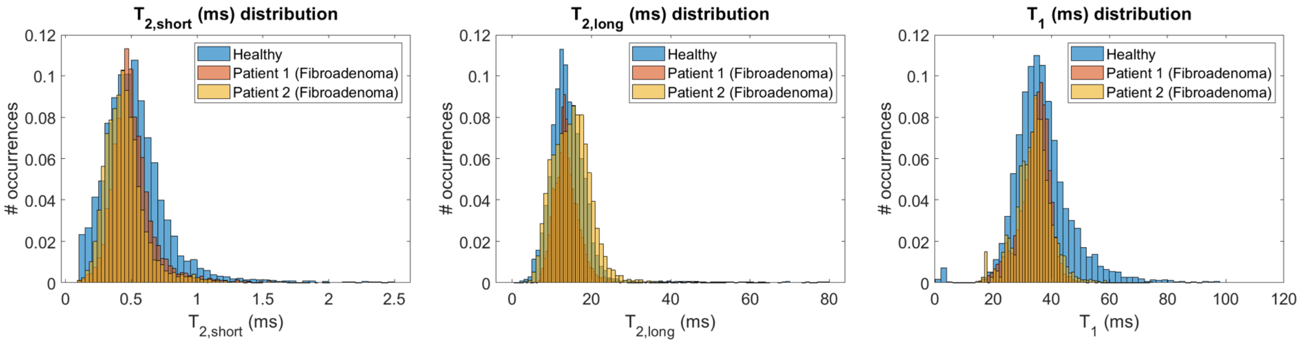


**Supplementary Figure S9.** Distributions of $^{23}$Na relaxation properties in the fibroglandular tissue of one healthy subject (light blue distribution) and two fibroadenoma patients (pink and yellow distributions). On the y-axis the number of occurrences is normalized by the total number of occurrences. The x-axis report, from left to right, $T_{2,s}$, $T_{2,l}$ and $T_1$ in ms. For all relaxation times the three distribution are in good agreement, showing little change in $T_1$, $T_{2,l}$ and $T_{2,s}$ in the two fibroadenoma patients compared to the healthy control. While a substantial change of relaxation times in the lesion would likely result in two separate peaks in the distributions, in both patients only a single peak is observed.

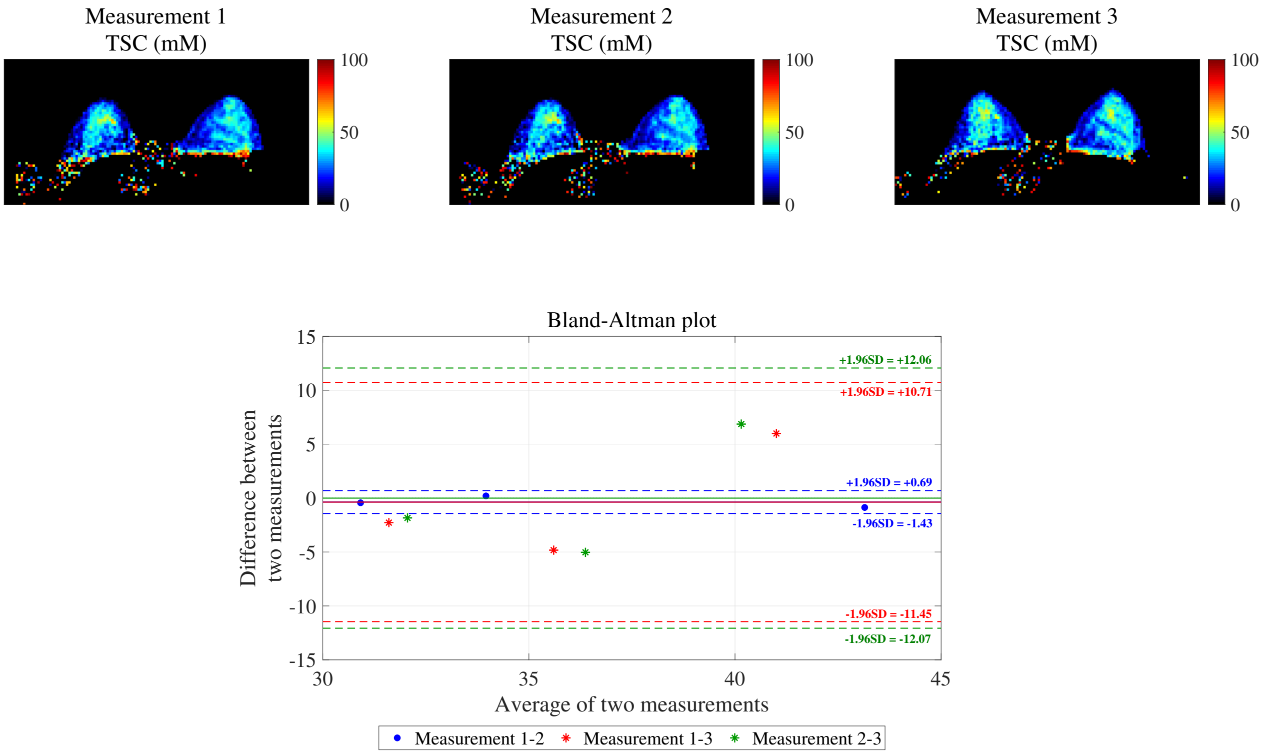


**Supplementary Figure S10.** TSC repeatability results. Top: TSC maps calculated from three separate measurements in a 27-years-old healthy subject. Measurements 1 and 2 were performed within the same session (inter-assay), ans measurement 3 was performed in a separate session (inter-assay). Bottom: Bland-Altman plot. For each pair of measurements (1-2, 1-3, 2-3) the difference between two measured TSC (mean in fibroglandular tissue) was plotted versus the average of the two measurements. Each color corresponds to a pair of measurements. The circles refer to measurements within the same session (intra-assay) while the stars refer to measurements performed in separate sessions (inter-assay). The difference between TSC acquired within the same session (measurement 1-2) was less than 1.17 standard deviations. The difference between TSC acquired in separate sessions (measurement 1-3 and measurement 2-3) was less than 1.13 standard deviations.

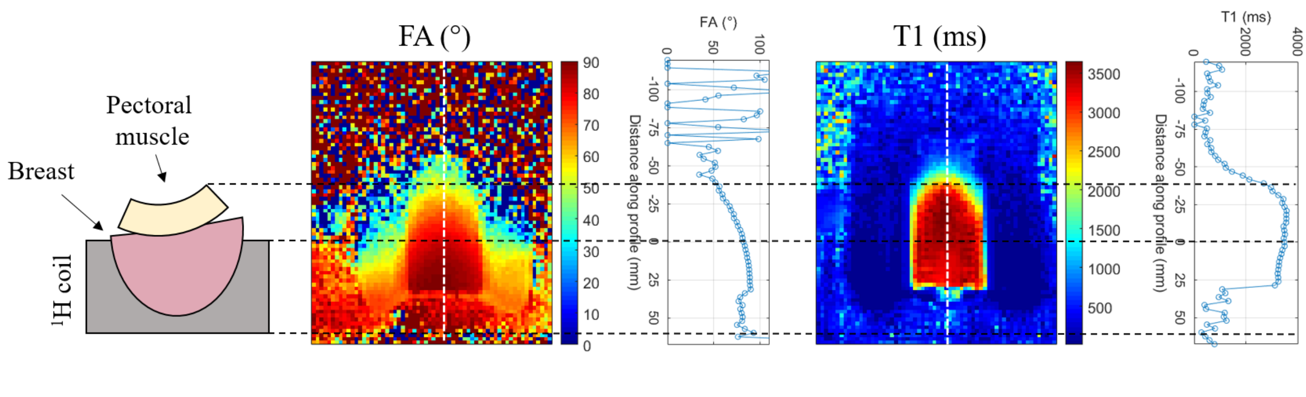


**Supplementary Figure S11.** Flip angle (FA) and $T_1$ maps in a water/oil phantom. For simplicity, unilateral experiments are shown. The schematic on the far left shows the location of the $^1$H Transmit/Receive volume coil, of the breast and of the pectoral muscle in the in vivo scenario. The horizontal dashed lines show the corresponding locations in the phantom in the experimental set-up. The phantom consisted of a inner bottle containing a water based mixture, immersed in an oil bath, and it was placed so that approximately half of it laid outside of the breast coil to emulate the position of the pectoral muscle in the in vivo condition. The 1D profiles along the white vertical dashed lines in the maps show the behaviour of FA and $T_1$ along the axis running along the length of the phantom. In the profile plots, the x-axis origin corresponds to the posterior limit of the coil and negative abscissas are outside of the coil (the pectoral muscle is typically located between 0 and -40 mm). While the FA rapidly decreases outside of the coil, $T_1$ is more uniform in the location corresponding to the pectoral muscle in the in vivo scenario. Specifically, $T_1$ is within 10% of its maximum between 0 and 32 mm outside of the coil and drops by 25% between 32 and 40 mm. This allows to select a large ROI covering most of the muscle tissue to robustly estimate its mean $T_1$.
